# Visceral adipose tissue and acute pancreatitis: a systematic review and meta-analysis

**DOI:** 10.7717/peerj.21254

**Published:** 2026-06-02

**Authors:** Hongfeng Sang, Xia Liu, Jichun Song

**Affiliations:** 1 General Surgery, Capital Medical University Xuanwu Hospital, Beijing, China; 2 School of Nursing, Capital Medical University Xuanwu Hospital, Beijing, China

**Keywords:** Visceral adipose tissue, Acute pancreatitis, Systematic review, Predictive value

## Abstract

**Background:**

Visceral adipose tissue (VAT) contributes to the severity of acute pancreatitis (AP) through systemic inflammation and metabolic dysregulation, but its predictive value remains unclear.

**Method:**

PubMed, Embase, Cochrane Library, and Web of Science databases were searched to collect related studies until April 9, 2025, and the literature search was updated as of January 30, 2026. The quality of the included studies was assessed using the NIH scale. Statistical analysis was performed using Stata 15.0.

**Result:**

The meta-analysis comprised 13 articles in total with 2,917 patients, and the overall quality of the included articles was high. The meta-analysis indicated high VAT content in patients with moderately severe AP (MSAP) (standardized mean difference (SMD) = 0.61, 95% confidence interval (CI) [0.12–1.11]) and patients with severe AP (SAP) (SMD = 0.85, 95% CI [0.17–1.52]) than that of patients with mild AP (MAP). No statistical difference in VAT content was found between patients with MSAP and SAP (SMD = 0.36, 95% CI [−0.03–0.75]). VAT was an independent risk factor for SAP (OR = 2.12, 95% CI [1.34–3.36]), with moderate diagnostic efficacy for AP (AUC = 0.74, 95% CI [0.63–0.85]).

**Conclusion:**

This systematic review suggests that VAT is associated with SAP and may have comparable diagnostic performance to established tools. However, these findings are based on observational studies with significant methodological limitations, including lack of blinding and substantial heterogeneity. Therefore, the evidence should be interpreted with caution, and the associations identified require confirmation in well-designed prospective studies incorporating rigorous blinding. VAT may serve as a potential reference for future precision assessment tools, but its clinical utility remains to be validated.

## Introduction

Acute pancreatitis (AP) is a common clinical gastrointestinal emergency, and its incidence has been rising annually worldwide. The latest data show that the global annual incidence of AP is about 34 cases per 100,000 people (Iannuzzi et al., 2022; Lee & Papachristou, 2019; Petrov & Yadav, 2019). AP is highly variable. Mild AP (MAP) can spontaneously resolve, while the mortality rate of severe AP (SAP) ranges from 20% to 30% (Tan et al., 2024). Long-term outcomes for SAP patients are concerning, with a high risk of mortality even after hospital discharge (Li et al., 2022; Selin et al., 2025). The long-term mortality rates remain higher in AP patients than in the general population, especially in cases of severe or non-biliary pancreatitis. Survivors often experience persistent organ dysfunction, including pancreatic insufficiency, diabetes, and chronic pancreatitis, which significantly affect their quality of life. Moreover, repeated episodes of severe AP can lead to the development of chronic pancreatitis, characterized by irreversible pancreatic fibrosis and progressive loss of pancreatic function. Chronic pancreatitis is a key risk factor for pancreatic cancer. It has been reported that patients with chronic inflammation and fibrosis are at significantly higher risk of developing pancreatic malignancies (Routh & Manickam, 2024; Swentek, Chung & Ichii, 2021; Szatmary et al., 2022). These findings highlight the multifaceted impact of SAP, not only on short-term survival but also on long-term health. Therefore, early detection and intervention of SAP are necessary. The underlying causes of AP are varied and multifactorial, and include factors such as gallstones, alcohol abuse, and hyperlipidemia. Obesity, which is closely associated with hyperlipidemia, is also an important contributing factor to AP and serves as a typical mechanism to show how metabolic disturbances can increase the risk of pancreatitis (Schepers et al., 2019). Obesity, especially disorders of fat metabolism, may further exacerbate pancreatic injury.

The underlying pathological process of AP involves the abnormal activation of pancreatic enzymes, leading to the autodigestion of pancreatic tissues. In individuals with obesity, excessive free fatty acids (FFAs) released from adipose tissue activate systemic inflammatory responses and lipotoxicity, which can directly impair pancreatic function (Wang et al., 2024b). Moreover, visceral adipose tissue (VAT), which surrounds the abdominal organs, differs from subcutaneous adipose tissue (SAT) in both functional and metabolic characteristics (Małodobra-Mazur et al., 2020). VAT is a metabolically active tissue that secretes various bioactive molecules, including FFAs, cytokines (*e.g.*, IL-6, TNF-α), and adipokines (*e.g.*, leptin), which play significant roles in inflammation, metabolism, and immune responses (Mohamed-Ali, Pinkney & Coppack, 1998; Singh et al., 2019; Weisberg et al., 2003). These molecules can enter the pancreas through the portal circulation, contributing to pancreatic dysfunction and systemic inflammation (Wang et al., 2025). The excessive secretion of inflammatory factors from VAT forms an adipokine storm, which can exacerbate the progression of AP.

Furthermore, VAT accumulation is associated with insulin resistance, which can activate pancreatic stellate cells *via* the TLR4/NF-κB pathway, promoting fibrogenesis and microvascular dysfunction (Catalán et al., 2024; Elkins et al., 2025; Engin, 2024; Li et al., 2023; Silva-Reis et al., 2024). Therefore, increased VAT content is strongly associated with metabolic disturbances and several acute diseases, including cardiovascular events and metabolic syndrome (Dal & Ulutas, 2021; Yang et al., 2025). In recent years, observational studies have found that increased VAT area (VATA) is positively associated with the incidence and severity of SAP (Saad et al., 2023). Possible mechanisms include excessive secretion of inflammatory factors from VAT to form an adipokine storm, while hypertriglyceridemia due to increased insulin resistance further increases the risk of pancreatic injury (Liao et al., 2024; Tian et al., 2024).

The visceral adiposity index (VAI), based on waist circumference, body mass index (BMI), triglycerides, and high-density lipoprotein cholesterol, is commonly used in clinical practice. While VAI is commonly used for assessing visceral fat, CT scanning remains the gold standard for directly measuring VAT, particularly through cross-sectional adipose tissue area measurements at the L3 vertebral level (Chan et al., 2023).

Currently, several models have been proposed to assist in early risk stratification in AP, including the Systemic Inflammatory Response Syndrome (SIRS) criteria and the Acute Physiology and Chronic Health Evaluation II (APACHE II) score, both of which primarily rely on early inflammatory and physiological disturbances. Although recently developed scoring systems, such as the Extra-Pancreatic Inflammation on CT (EPIC) score and the Pancreatitis Activity Scoring System (PASS), have used to improve early prognostication, their accuracy and consistency remain suboptimal (Chen et al., 2017). Therefore, it is imperative to explore additional reliable indicators to support early and precise assessment of AP severity. VAT has been reported to play a crucial role in metabolic diseases like cardiovascular disease and diabetes, which may influence the clinical outcomes of AP patients. Nonetheless, it has not been adequately incorporated into prediction models. Some studies have shown that fat distribution, including both subcutaneous and visceral fat, can impact the severity of AP (Capurso et al., 2023).

Therefore, this systematic review and meta-analysis aimed to quantify the association between VATA or VAI and AP, particularly severe AP (SAP). Specifically, we combined the odds ratios (OR) for VAT as a risk factor for AP and the areas under the curve (AUC) for VAT as a diagnostic marker. The aim of this study is to clarify the clinical value of VAT as a supplementary biomarker for early risk assessment, diagnostic assessment, and preventive management in patients with AP.

## Method

This study was reported according to the Preferred Reporting Items for Systematic Reviews and Meta-Analyses (PRISMA) guidelines (Appendix checklist). The study protocol was registered with PROSPERO (CRD420250651237).

### Search strategy

Pubmed, Embase, Cochrane Library, and Web of Science databases were searched to collect related studies from database inception to April 9, 2025, without language restrictions. The search strategy was designed by combining “acute pancreatitis” and “visceral fat” as subject terms and their free terms like boolean logic. The literature search was updated on January 30, 2026, to include the most recent relevant studies. The detailed search results are shown in Table S1.

### Selection criteria

Inclusion criteria:

(1) Participants were adults (≥18 years) diagnosed with AP, with a clear diagnosis process and basis during the hospital admission. AP cases included patients with mild AP (MAP), moderately severe AP (MSAP), and SAP, as well as those with hyperlipidemic AP. AP was diagnosed when at least two of the following three criteria were met: (1) acute pain in the upper abdomen; (2) an increase in serum amylase or lipase; and (3) characteristic imaging findings of AP, in accordance with the Japanese guideline for the management of AP. The severity of AP was categorized into three levels: MAP, MSAP, and SAP. MAP was defined as the absence of organ failure and local or systemic complications. MSAP was defined as the presence of transient organ failure (<48 h) and/or local or systemic complications. SAP was defined as persistent organ failure lasting >48 h.

(2) Studies must clearly report the measurement process of VAT-related parameters, including: (a) VATA quantified by CT scans (reported as cross-sectional VATA at a specified vertebral level) and (b) VAT-related indices derived from body composition or anthropometric measures, such as VAI. In this review, both VATA and VAI were considered as VAT-related outcomes, and subgroup analyses were conducted based on the type of VAT measurement employed.

(3) The study design was an observational study.

(4) Studies must provide relevant data for the calculation of indices, such as AUC values and OR. The results of the OR can be analyzed separately in the later stages based on whether the independent variables were continuous or categorical.

Exclusion criteria: (1) patients with chronic or recurrent pancreatitis, pregnant or lactating women, or patients with a hospital stay of less than 48 h; (2) relevant data not consistent with the study purpose; (3) systematic evaluation, reviews, conference abstracts, animal experiments, case reports, letters, or comments.

### Study selection

Studies searched from the databases were exported to EndNote 21.0. Then, duplicates were ruled out (April 10, 2025). Two independent researchers (Sang HF and Liu X) screened the titles and abstracts and then read the full texts of potentially eligible studies to determine eligible studies. Subsequently, the two researchers cross-checked the included studies. If there was a dispute, a third reviewer (Jin J) was consulted to reach a consensus.

### Data extraction

Two researchers (Sang HF and Liu X, May 4, 2025) independently extracted relevant data from the included studies. The extracted information included first author, publication year, country, disease severity, sample size, basic information about the patients, and VAT-related indexes. Finally, the extracted information was cross-checked by the two researchers. If there were any dissents, they negotiated and reached a consensus.

### Risk of bias assessment

The quality of the included studies was assessed independently by two researchers (Sang HF and Liu X). Disagreements, if any, were addressed by a third researcher (Jin J). The risk of bias was assessed using the National Institutes of Health (NIH) Quality Assessment Tool for Observational Cohort and Cross-Sectional Studies. This instrument comprises 14 evaluation items. Each item was answered with “yes,” “no,” or “other,” based on the rigor of the study’s design and implementation. A total score of 0–5 was indicative of a high risk of bias (poor quality), 6–10 represented a moderate risk of bias (fair quality), and 11–14 denoted a low risk of bias (good quality).

### Data synthesis

Stata 15.0 was used for statistical analysis. Continuous data were expressed as the standardized mean differences (SMD) with 95% confidence interval (CI). An SMD of 0.2−0.5 was considered as a small effect, 0.5−0.8 as a medium effect, and >0.8 as a large effect. In each study, the odds ratio (OR) and 95% CI derived from the maximum-adjustment model were used as effect sizes. If studies reported the area under the curve (AUC) values for diagnostic efficacy, the following formula was used for meta-analysis: AUC ± 1.96*sqrt (sample size) (Gemelli et al., 2025). Heterogeneity was assessed using I^2^. If I^2^ ≤ 50%, a fixed-effects model was selected. Otherwise, a random-effects model was used. Sensitivity analyses were performed to determine the stability of the results. Subgroup analyses and meta-regression were performed to ascertain potential sources of heterogeneity. For outcome measures reported in more than 10 articles, publication bias was evaluated using funnel plots and Egger’s test. For all data analyses, *P* < 0.05 was considered statistically significant, except for the Egger test, where a *P* value > 0.05 implied no publication bias.

## Result

### Results of literature search and screening

**Figure 1:**
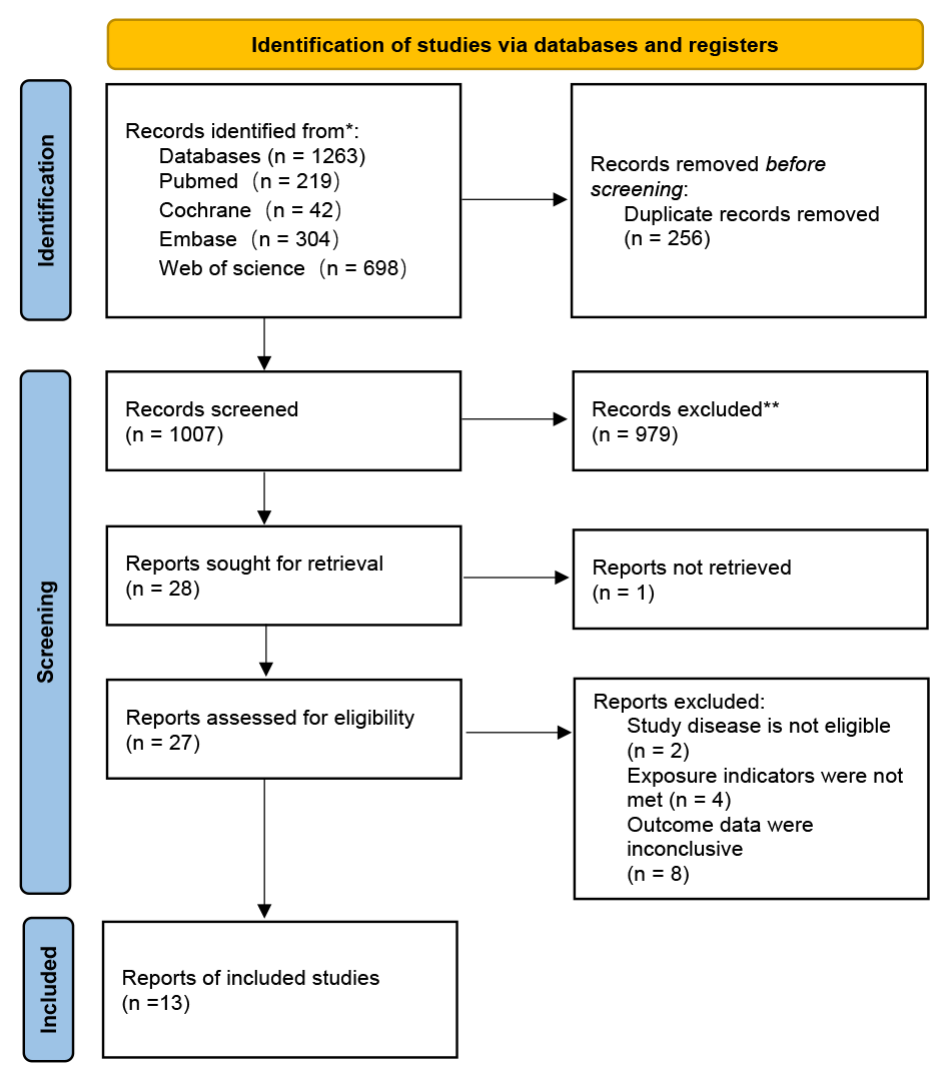
PRISMA flow diagram.

A total of 1,263 relevant articles were retrieved from four databases. After 256 duplicates were excluded, 1,007 articles remained. After reviewing the titles and abstracts, 979 articles were removed. The full texts of the remaining 28 articles were reviewed to assess eligibility. The full text of one article was inaccessible. Two articles were excluded because their research aims did not match the disease of interest. Four articles were excluded because they used different exposure measures or assessment methods that did not align with our predefined criteria. Eight articles were excluded because their reported outcome indicators did not match the outcomes of interest in our study. The supplementary search did not find any additional articles that met the inclusion criteria. Finally, 13 studies were included (Fig. 1).

The 13 eligible articles (Akturk, Ozbal Gunes & Hekimoglu, 2021; Angadi et al., 2024; Beydogan et al., 2021; Higaki et al., 2021; Huang et al., 2022; Madico et al., 2019; Sternby et al., 2019; Wang et al., 2024a; Xia et al., 2022; Xie et al., 2019; Yoon et al., 2017; Zhou et al., 2021; Zhu et al., 2024) involved 2,917 patients, including 554 patients with SAP. These studies were published in China, Europe, Japan, Korea, India, Turkey, and France. The age of patients ranged from 26 to 80 years old, and BMI ranged from 19.48 to 32.57 kg/m^2^ (Table 1).

**Table 1:** Characteristics of included studies.

Author	Year	Country	Sample size	Age	BMI	Measurement index	Measurement method	Measurement time	Disease grouping (N)		
Huang et al.	2022	China	308	45.37 ± 13.74	25.00 ± 4.93	VAI	Index Calculation	Upon Admission	MAP (186)	MSAP (60)	SAP (62)
Zhu et al.	2024	China	283	40.73 ± 9.33	25.89 ± 3.63	VAI	Index Calculation	Upon Admission	MAP (152)	MSAP to SAP (131)	
Sternby et al.	2019	Europe multicentre	454	50.65 ± 20.08	27.88 ± 4.69	VAT(area)	CT(L3)	Upon Admission	MAP (163)	MSAP (225)	SAP (66)
Higaki et al.	2021	Japan	119	62.00 ± 18.01	22.48 ± 3.00	VFA(area)	CT(L3)	Upon Admission			SAP (119)
Zhou et al.	2021	China	392	55.05 ± 23.07	25.19 ± 4.14	VAT(area)	CT(L3)	NI	MAP (178)	MASP (161)	SAP (53)
Yoon et al.	2017	Korea	203	53.30 ± 17.90	24.15 ± 3.93	VAT(area)	CT(L3)	Upon Admission	MAP (128)	MSAP (62)	SAP (13)
Xie et al.	2019	China	306	50.60 ± 18.70	24.40 ± 4.50	VAT(area)	CT(L3-L4)	After Admission within 24 h	MAP (204)	MSAP (56)	SAP (46)
Xia et al.	2022	China	227	40.50 ± 9.60	25.99 ± 3.46	VAI	Index Calculation	Upon Admission	MAP (125)	MASP (80)	SAP (22)
Wang et al.	2024a	China	158	48.40 ± 19.45	26.36 ± 4.34	VAT(area)	CT(L2-L3)	Upon Admission		MASP (102)	SAP (56)
Beydogan et al.	2021	Turkey	174	58.58 ± 18.24	NI	VAT(area)	CT(L2-L3)	Upon Admission	MAP (142)	MSAP (32)	
Angadi et al.	2024	India	74	38.98 ± 12.97	25.26 ± 2.82	VAT(area)	CT(L2-L3/L-L4)	Upon Admission	MAP/MSAP (37)		SAP (37)
Akturk, Ozbal Gunes & Hekimoglu	2021	Turkey	107	55.74 ± 17.19	NI	VAT(area)	CT(L3)	Upon Admission	MAP (41)	MSAP (41)	SAP (25)
Madico et al.	2019	France	112	56.3 ± 21.6	26.3 ± 6.9	VAT(area)	CT(L4-L5)	Upon Admission	MAP (57)		SAP (55)

**Notes.**

BMI body mass index MAP mild acute pancreatitis MSAP moderately severe acute pancreatitis NI no information SAP severe acute pancreatitis VAI visceral adiposity index VAT visceral adipose tissue VFA visceral fat area

### Risk of bias assessment

The overall quality of the included studies was good. Most studies clearly reported study objectives and populations, controlled exposure and confounders, and specified the measurement methods of outcomes. In addition, the data in most studies were complete. The dropout rates of most studies were less than 20%, which was considered as part of the quality evaluation. Although most studies reported measurements of repeated exposure factors, some did not. Blinding of outcome assessors was not mentioned in any of the studies (Table S2).

### Meta-analysis

#### MAP and MSAP

**Figure 2:**
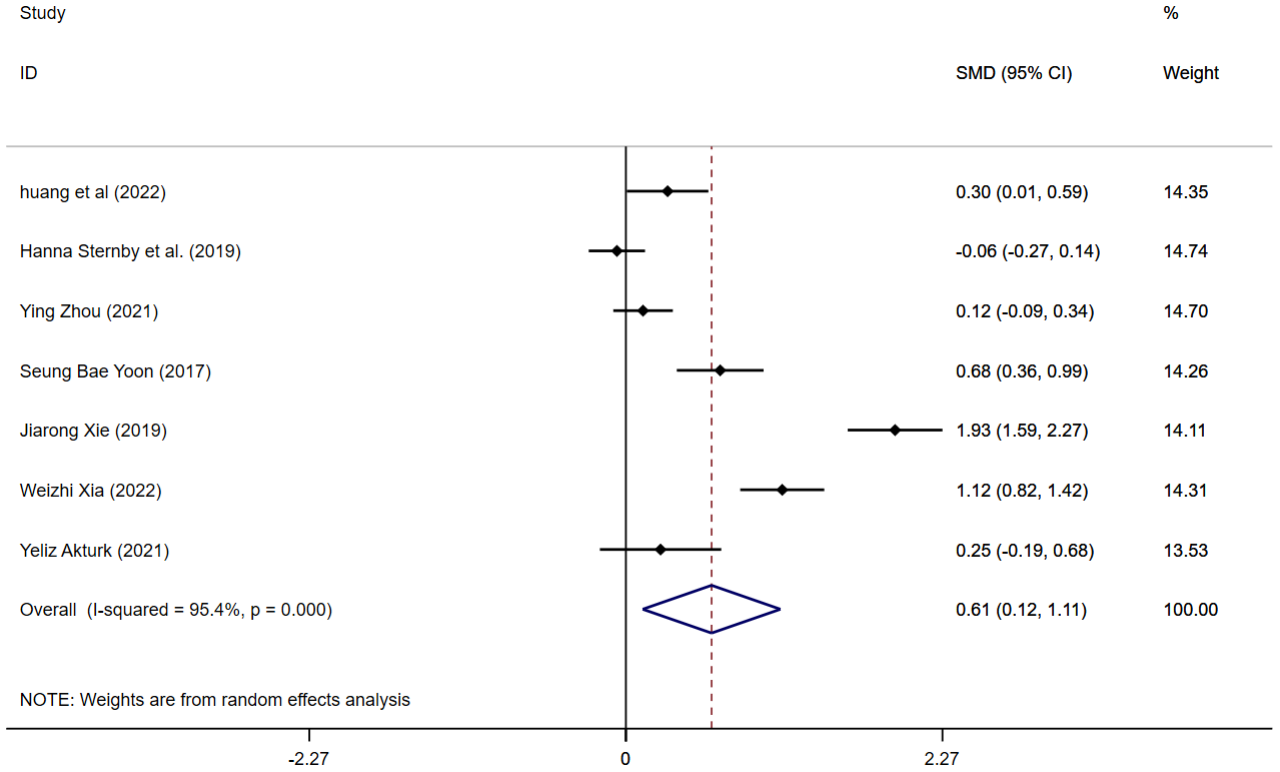
Forest plot of MAP *vs* MSAP.

Seven articles reported VAT content in MAP and MSAP. The meta-analysis indicated that MSAP patients had greatly higher VAT content than MAP patients (SMD = 0.61, 95% CI [0.12–1.11], *I*^2^ = 95.4%) (Fig. 2), with high heterogeneity. Sensitivity analysis suggested robust findings, and no source of heterogeneity was identified (Fig. S1). Subgroup analyses were further conducted based on measurement methods and geographical regions. When studies were stratified by measurement method, the pooled effect size was 0.71 (95% CI [−0.10–1.51]) for VAI and 0.58 (95% CI [−0.07–1.22]) for VATA (Fig. S2). Subgroup analysis by region revealed that the pooled effect size was 0.86 (95% CI [0.08–1.64]) for studies conducted in China, −0.06 (95% CI [−0.27–0.14]) in Europe, 0.68 (95% CI [0.36–0.99]) in Korea, and 0.25 (95% CI [−0.19–0.68]) in Turkey (Fig. S3). The meta-regression analysis revealed that age, BMI, and measurement method were not the sources of heterogeneity (Table S3). We conducted a separate meta-analysis for the VATA results, and the analysis showed WMD = 18.97 (95% CI [2.81–35.12]) cm^2^ (Fig. S4).

#### MAP and SAP

**Figure 3:**
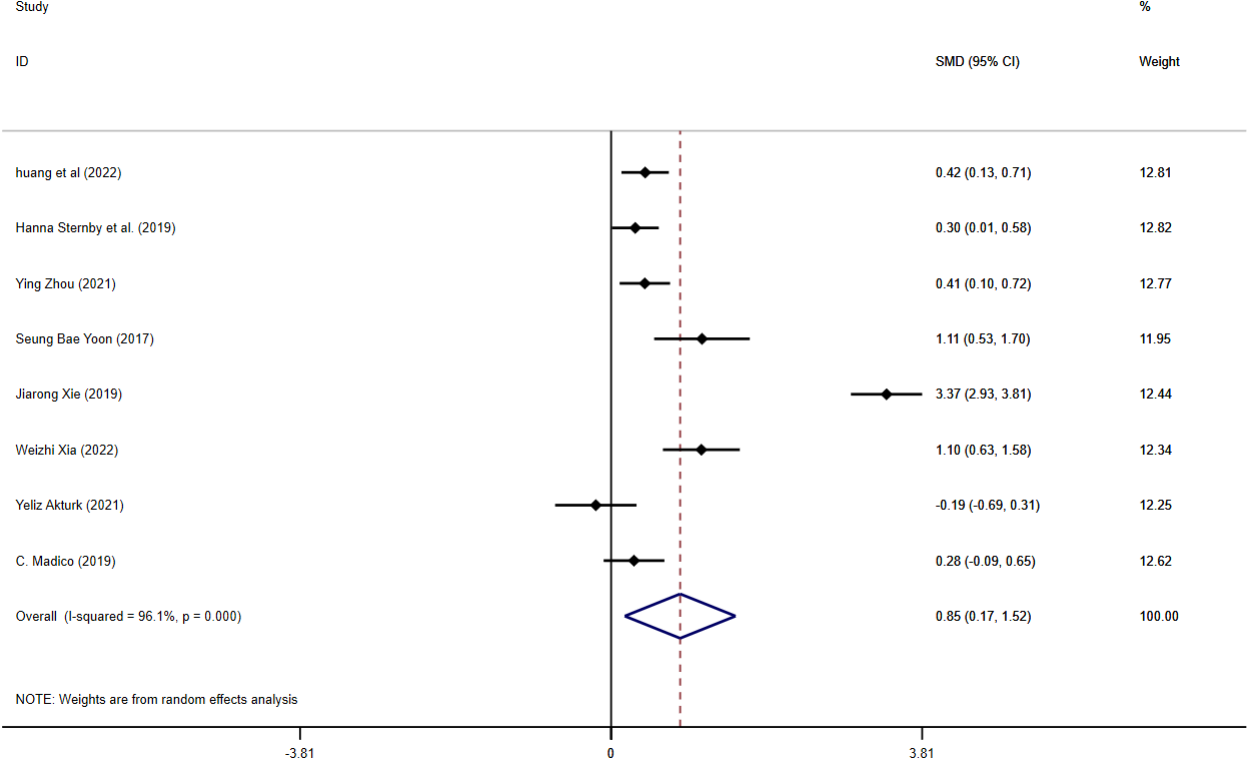
Forest plot of MAP *vs* SAP.

Seven articles reported VAT content in MAP and SAP. The meta-analysis revealed that SAP patients had greatly higher VAT content than MAP patients (SMD = 0.85, 95% CI [0.17–1.52], *I*^2^ = 96.1%) (Fig. 3), with high heterogeneity. Sensitivity analyses indicated robust findings, and no source of heterogeneity was identified (Fig. S5). To further ascertain potential sources of variability, subgroup analyses were performed based on measurement methods and geographic regions. When studies were stratified by measurement method, the pooled effect size was 0.73 (95% CI [0.06–1.41]) for VAI and 0.88 (95% CI [0.17–1.52]) for VATA (Fig. S6). Subgroup analysis by region revealed that the pooled effect size was 1.32 (95% CI [0.07–2.57]) for studies conducted in China, 0.30 (95% CI [0.01–0.58]) in Europe, 1.11 (95% CI [0.53–1.70]) in Korea, −0.19 (95% CI [−0.69–0.31]) in Turkey, and 0.28 (95% CI [−0.09–0.65]) in France (Fig. S7). Meta-regression analysis did not identify sources of heterogeneity (Table S3). We conducted a separate meta-analysis for the VATA results, and the analysis showed WMD = 33.42 (95% CI [5.35–61.48]) cm^2^ (Fig. S8).

#### MSAP and SAP

**Figure 4:**
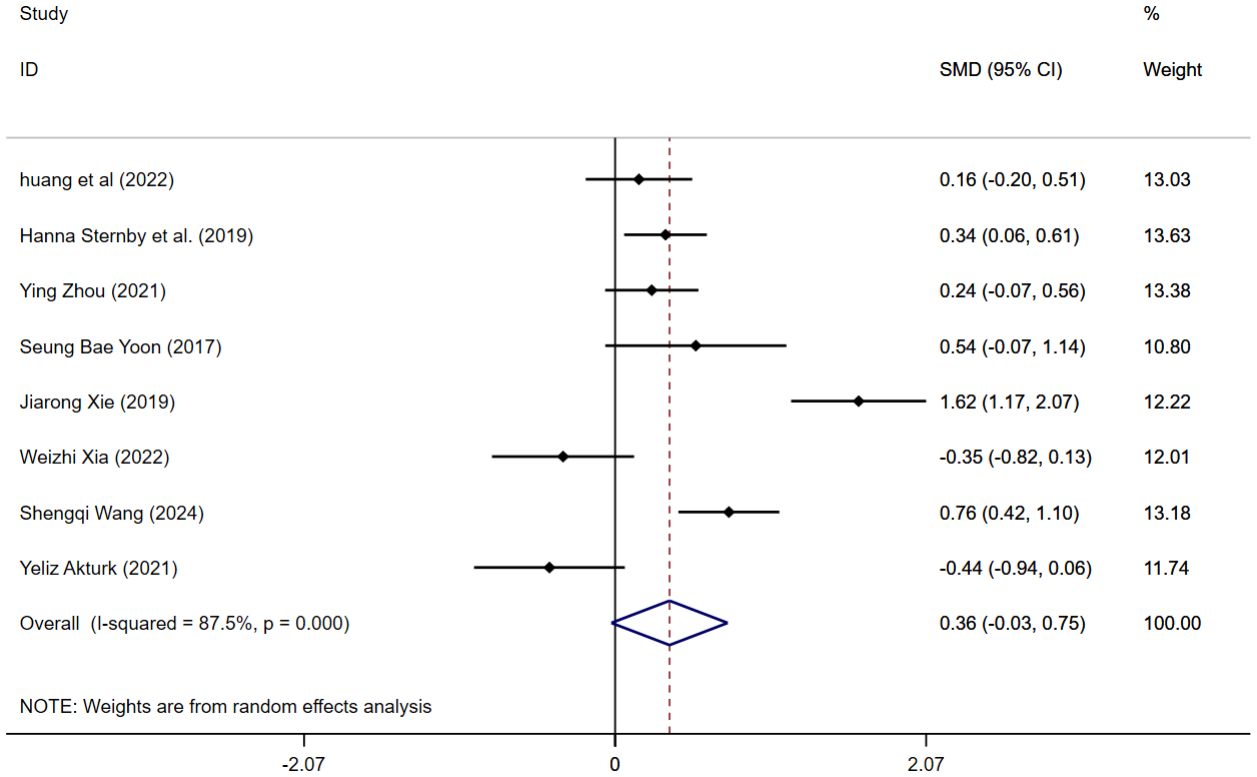
Forest plot of MSAP *vs* SAP.

Eight articles reported VAT content in MSAP and SAP. The meta-analysis indicated no difference in VAT content between MSAP and SAP (SMD = 0.36, 95% CI [−0.03–0.75], *I*^2^ = 87.5%) (Fig. 4), with high heterogeneity. Sensitivity analyses suggested robust findings, and no source of heterogeneity was identified (Fig. S9). Subgroup analyses were performed according to measurement methods and geographic regions. When studies were stratified by measurement method, the pooled effect estimate was −0.07 (95% CI [−0.56–0.42]) for VAI, and 0.51 (95% CI [−0.03–0.75]) for VATA (Fig. S10). The subgroup analysis by region revealed that the pooled effect estimate was 0.49 (95% CI [−0.07–1.05]) for studies conducted in China, 0.34 (95% CI [0.06–0.61]) in Europe, 0.54 (95% CI [−0.07–1.14]) in Korea, and −0.44 (95% CI [−0.94–0.06]) in Turkey (Fig. S11). The meta-regression analysis by age, BMI, and indicator measurements showed that none of them was a source of heterogeneity (Table S3). We conducted a separate meta-analysis for the VATA results, and the analysis showed WMD = 17.63 (95% CI [−3.47–38.73]) cm^2^ (Fig. S12).

#### MAP and MSAP-SAP

**Figure 5:**
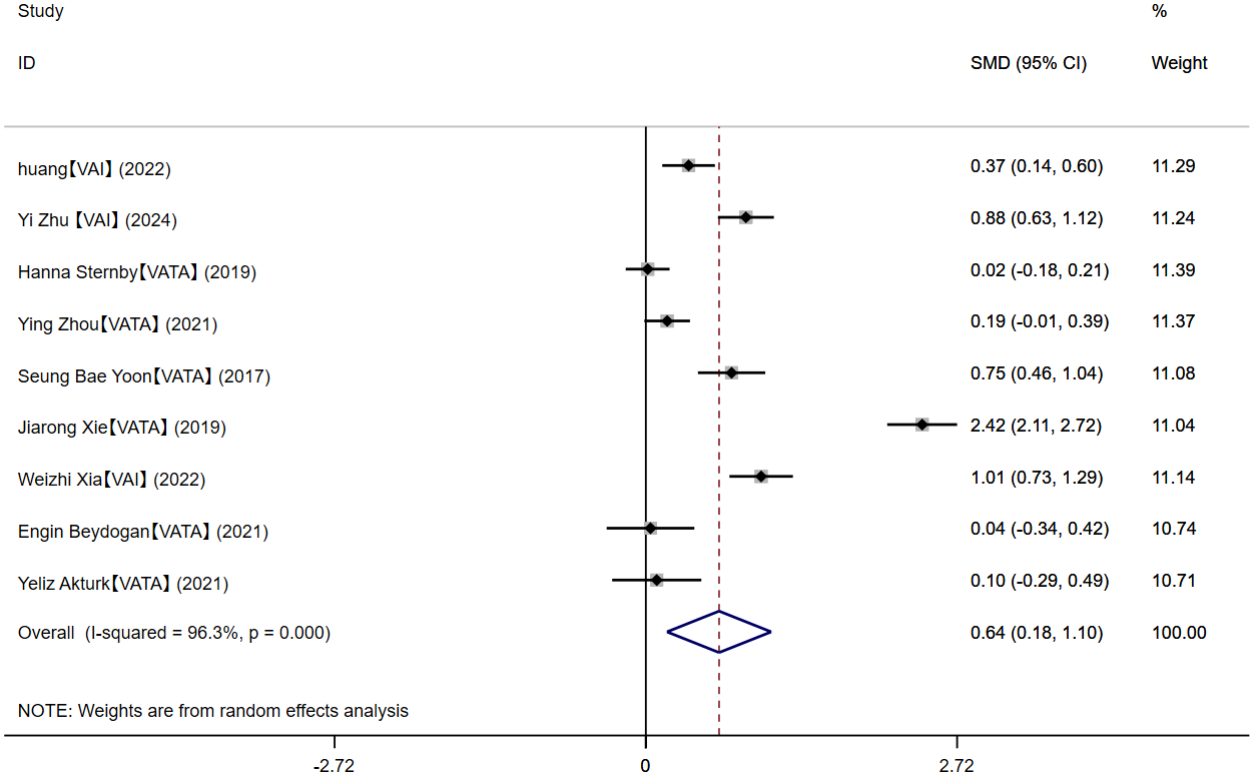
Forest plot of MAP *vs* MSAP-SAP.

Ten relevant articles were included in the analysis, and the meta-analysis evinced that MSAP-SAP showed higher VAT content than MAP (SMD = 0.64, 95% CI [0.18–1.10], *I*^2^ = 96.3%) (Fig. 5), with high heterogeneity. Sensitivity analyses indicated relatively robust results, and no source of heterogeneity was identified (Fig. S13). The subgroup analysis by measurement method demonstrated that the pooled effect estimate was 0.75 (95% CI [0.36–1.13]) for VAI and 0.58 (95% CI [−0.11–1.28]) for VATA (Fig. S14). Subgroup analysis by region showed that the pooled effect estimates were 0.97 (95% CI [0.27–1.66]) for studies published in China, 0.75 (95% CI [0.46–1.04]) in Korea, 0.02 (95% CI [−0.18–0.21]) in Europe, and 0.07 (95% CI [−0.21–0.34]) in Turkey (Fig. S15). Meta-regression analyses elicited that age, BMI, and measurement methods were not sources of study heterogeneity (Table S3). We conducted a separate meta-analysis for the VATA results, and the analysis showed WMD = 21.21 (95% CI [1.84–40.59]) cm^2^ (Fig. S16).

#### MAP-MSAP and SAP

**Figure 6:**
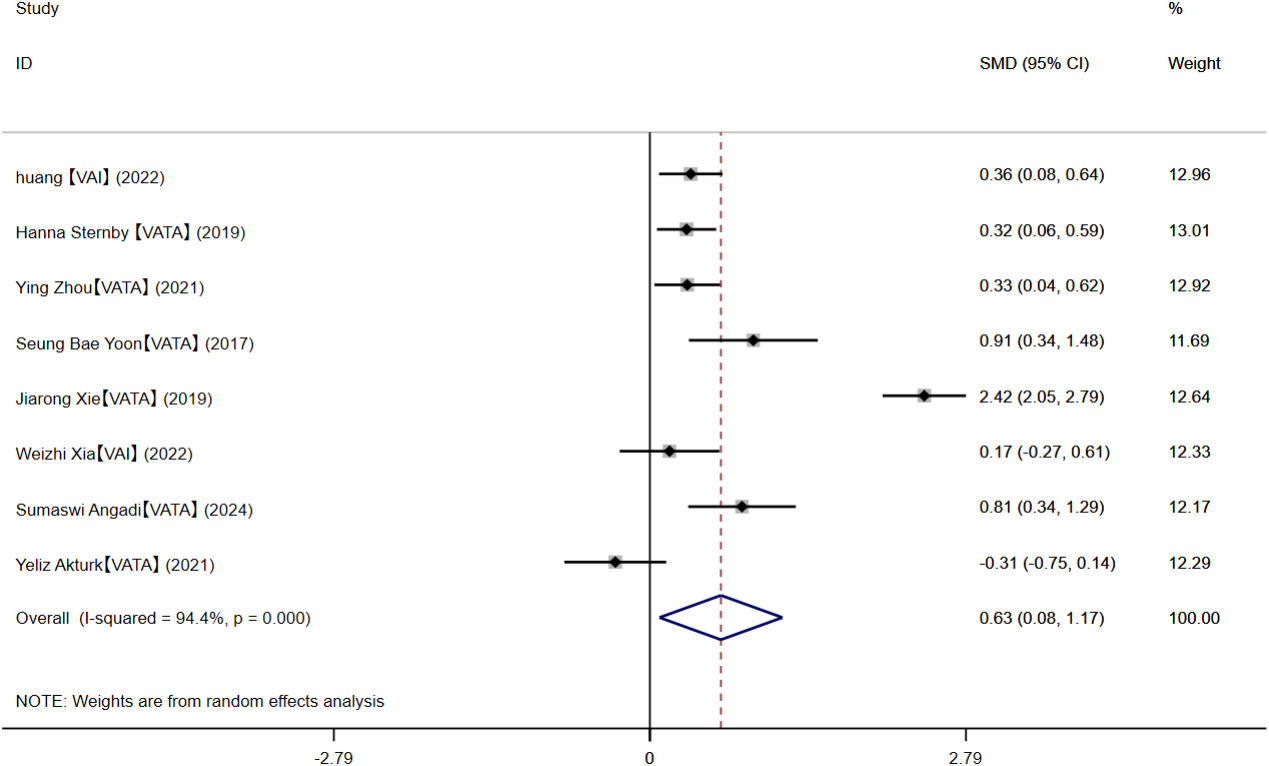
MAP-SMAP VS SAP.

Eight studies compared VAT content in MAP-MSAP and SAP patients. The pooled results indicated that patients with SAP showed higher VAT levels (SMD = 0.63, 95% CI [0.08–1.17]; *I*^2^ = 94.4%) (Fig. 6), although substantial heterogeneity was observed. Sensitivity analysis indicated robust findings (Fig. S17). Subgroup analysis by measurement methods revealed that the effect size was 0.31 (95% CI [0.07–0.54]) for VAI and 0.75 (95% CI [0.01–1.49]) for VATA (Fig. S18). The subgroup analysis by region indicated that the pooled effect sizes were 0.82 (95% CI [−0.15–1.79]) for China, 0.32 (95% CI [0.06–0.59]) for Europe, 0.91 (95% CI [0.34–1.48]) for Korea, 0.81 (95% CI [0.34–1.29]) for India (Fig. S19), and −0.31 (95% CI [−0.75–0.14]) for Turkey. Meta-regression found that age, BMI, and measurement methods were not the source of heterogeneity (Table S3). We conducted a separate meta-analysis for the VATA results, and the analysis showed WMD = 29.79 (95% CI [5.36–54.22]) cm^2^ (Fig. S20).

#### Diagnostic performance

**Figure 7:**
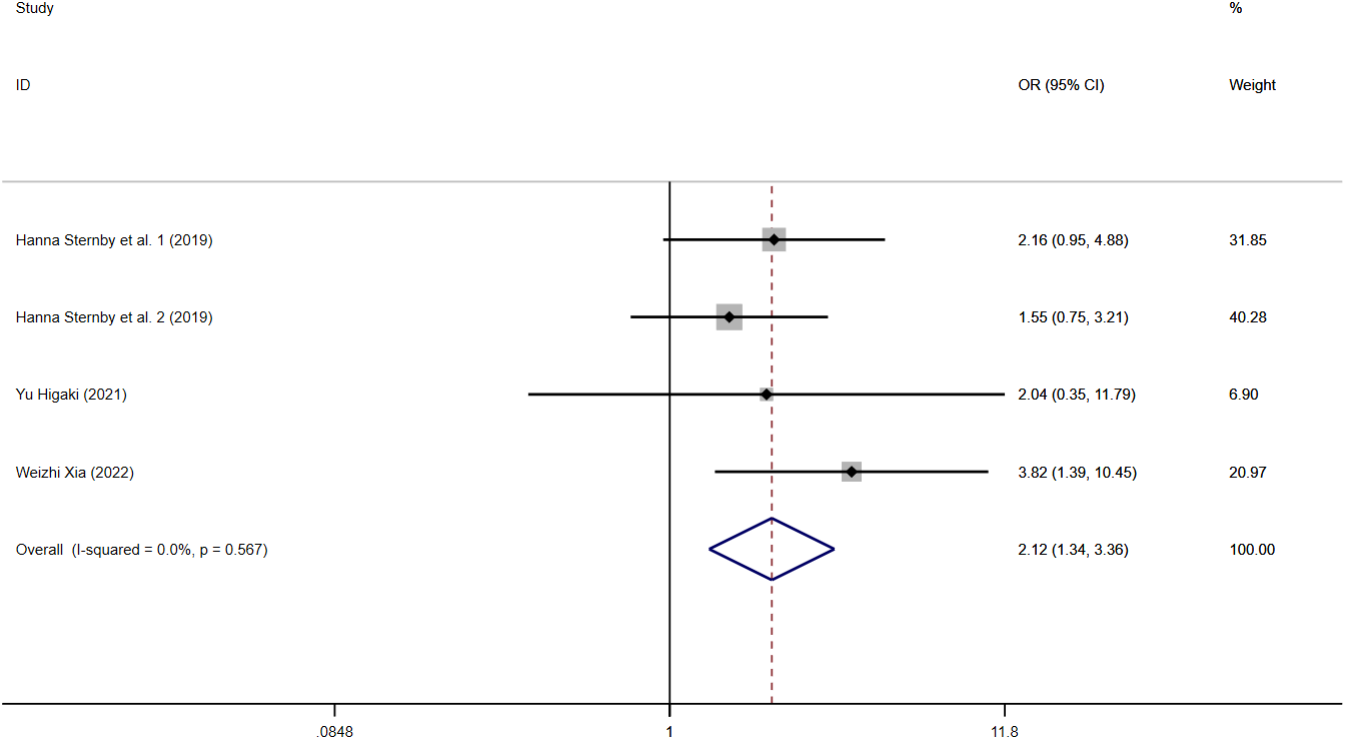
Forest plot of SAP(OR).

Three articles assessed SAP through multivariate adjustment. The meta-analysis suggested that VAT content was an independent risk factor for SAP (OR = 2.12, 95% CI [1.34–3.36], I^2^ = 0%) (Fig. 7). Sensitivity analyses showed robust findings, and no source of heterogeneity was identified (Fig. S21). The subgroup analysis by measurement method demonstrated that the pooled effect estimate was 3.82 (95% CI [1.39–10.45]) for VAI and 1.81 (95% CI [1.08–3.05]) for VATA (Fig. S22). The subgroup analysis by region revealed that the pooled effect estimate was 3.82 (95% CI [1.39–10.45]) for studies conducted in China, 1.79 (95% CI [1.04–3.09]) in Europe, and 2.04 (95% CI [0.35–11.79]) in Japan (Fig. S23). One article (Zhou et al., 2021), which used VAT as a continuous variable in a logistic regression model, was not included in the meta-analysis. The study indicated that for each unit increase in VAT, the risk of the disease increased by 1.003 (95% CI [1.00–1.006]). Another study (Huang et al., 2022), which used VAT as a categorical variable, reported an OR of 2.38 (95% CI [1.11–5.10]) (Table S4).

**Figure 8:**
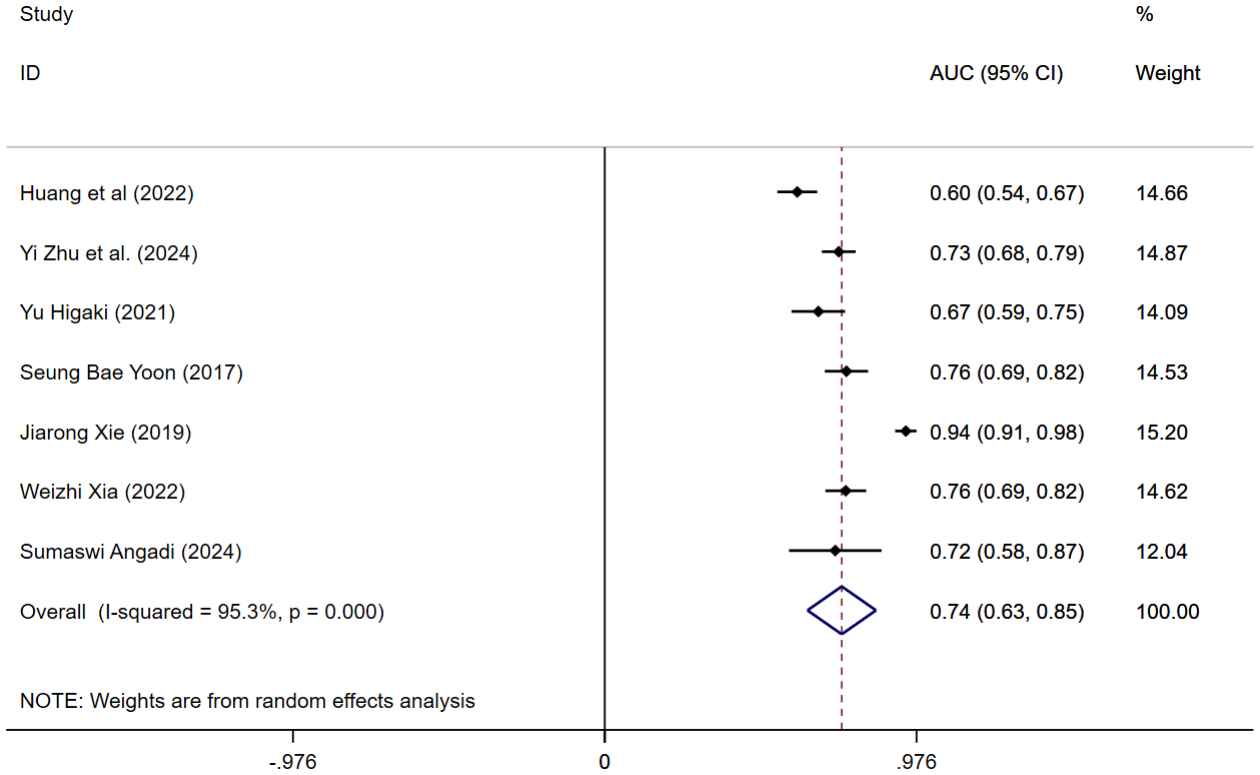
Forest plot of MSAP-SAP(AUC).

A meta-analysis of seven studies demonstrated that VAT has a moderate diagnostic performance for distinguishing AP patients, with an AUC of 0.74 (95% CI [0.63–0.85]; *I*^2^ = 95.3%), although substantial heterogeneity was observed (Fig. 8). Sensitivity analysis confirmed the robustness of the findings, and no clear source of heterogeneity was identified (Fig. S24). Subgroup analysis by measurement method showed an AUC of 0.70 (95% CI [0.61–0.79]) for VAI and 0.78 (95% CI [0.63–0.93]) for VATA, indicating that both metrics have moderate discriminative power for AP severity (Fig. S25). Further subgroup analysis based on disease severity showed that the AUC was 0.60 (95% CI [0.54–0.67]) for non-MAP, 0.74 (95% CI [0.70–0.78]) for hyperlipidemic AP, 0.68 (95% CI [0.61–0.76]) for SAP, and 0.85 (95% CI [0.67–1.03]) for MSAP-SAP (Fig. S26).

## Discussion

This systematic review and meta-analysis included 13 articles involving 2,917 patients to explore the relationship between VAT content and AP severity. Our results demonstrated that VAT content was higher in both MSAP and SAP patients compared to MAP patients.VAT had a moderate diagnostic ability for distinguishing between different severity levels of AP. This result suggests the vital role of VAT in the progression of AP. The underlying mechanisms include metabolic disorders, inflammatory responses, and anatomical compensations. The subgroup analyses indicated that both VAI and VATA measurements showed higher VAT content in more severe forms of AP. Specifically, the subgroup analysis by measurement method revealed that the pooled effect size for VAI was slightly higher than that for VATA in both MSAP and SAP, suggesting that the two methods may yield similar, but not identical, results. However, the effect sizes for both methods were relatively similar, indicating that both VAI and VATA can be used as valuable indicators of VAT content in AP patients.

According to epidemiological evidence, elevated levels of VAT, which increase the inflammatory cascade, are strongly associated with AP severity. In a prospective study, VAI is significantly and positively associated with the incidence of MSAP and SAP, and the risk of pancreatic necrosis is elevated 3.2-fold when VAI exceeds 2.5 (Jin et al., 2017). In addition, quantitative CT assessment reveals that those with a VATA > 100 cm^2^ have a notably increased risk of complications of infection, multiple organ dysfunction, acute peripancreatic fluid collection, and acute necrotic collection during hospitalization (Ji et al., 2019). Notably, VAI, as a composite index that includes body fat distribution and lipid metabolism indicators, better reflects the metabolic inflammatory status of an individual than traditional indicators such as BMI (Ferreira et al., 2019). It is closely associated with cardiovascular disease, diabetes mellitus, and non-alcoholic fatty liver disease, further suggesting its common pathologic pathway in inflammatory diseases (Deng et al., 2024; Wang et al., 2015; Wu et al., 2025). Particularly, among individuals with high VAI, inflammatory diseases are more common in young men, which may be related to the susceptibility to VAT accumulation due to poor lifestyles (such as high-fat diet, alcohol abuse, sedentary behavior). In addition, VATA is positively correlated with systemic inflammation, confirming that VAT promotes multi-system involvement (Liao et al., 2024). These findings emphasize the critical role of VAT in the progression of AP, particularly in more severe forms of AP. Given the strong association between VAT and AP severity, our results suggest that VAT may serve as a valuable indicator of AP and could aid in identifying patients at higher risk for complications.

While the results of this study are promising, it is important to discuss whether the difference in VAT between AP groups is clinically meaningful. Our analysis indicates that patients with SAP have significantly higher VAT compared to MAP patients (SMD = 0.85, 95% CI [0.17–1.52]). While this statistical difference is significant, the clinical significance of such a difference should be further explored. Specifically, future studies should identify clinical thresholds of VAT accumulation for the progression of AP severity, promoting the development of early interventions and improving patient outcomes.

Currently, prognostic assessment of AP in clinical practice relies primarily on scoring systems like SIRS, APACHE II, Ranson, and BISAP, which have limited accuracy, largely rely on laboratory parameters, and are less accurate for the early stage of the disease (Di et al., 2016). Furthermore, evidence of the incremental predictive value of most scoring systems in actual clinical practice is lacking. In contrast, our study found that VATA demonstrated superior predictive performance (AUC = 0.78) compared to conventional anthropometric indicators (VAI, AUC = 0.70). The diagnostic efficacy of VATA also approximated, or even exceeded, that of SIRS (AUC = 0.75) and APACHE II (AUC: 0.65–0.75) (Chen et al., 2017; Gu, Shang & Wang, 2025). Therefore, VAT may be a potentially valuable tool for the severity of AP. CT imaging is routinely performed upon hospital admission to assess pancreatic morphology and complications. From CT imaging, VAT measurements can be derived simultaneously, without additional burden or cost. Hence, it appears to be an accessible, objective, and reproducible tool for early risk stratification. Compared with traditional scoring systems, VAT reflects systemic inflammatory status and captures critical aspects of metabolic and immune dysregulation, which may be more directly linked to the underlying pathophysiology of AP. Accordingly, VAT may serve as a comprehensive risk factor and holds promise as an integral component of future precision assessment models.

In clinical practice, identifying and evaluating VAT accumulation is valuable for early risk assessment and intervention in AP patients. VAT not only serves as an early indicator of high-risk patients but also plays a crucial role in distinguishing between different severity levels of AP. Our study demonstrated that VATA had a favorable performance in discriminating severe forms of AP, such as SAP, compared to MAP. Specifically, the AUC values of VAT were higher in SAP (AUC = 0.68) and MSAP-SAP (AUC = 0.85), indicating its strong diagnostic performance in more severe cases. These results suggest that VAT is helpful in identifying patients at risk of complications and facilitating early interventions. On the one hand, VAT, as an early risk indicator, can assist in the traditional scoring system in identifying high-risk groups. On the other hand, the metabolic-immune-inflammatory imbalance reflected by VAT provides a new entry point for clinical intervention (Hotamisligil, 2006). For example, early intensive nutritional support, lipid metabolism modulation, or anti-inflammatory interventions may be considered for those with significant VAT accumulation to reduce the pro-inflammatory load of adipose sources (Barbosa et al., 2024; Lankila et al., 2025). In recent years, novel VAT-related indices such as VAI, CVAI, and LAP have been included in models for predicting AP severity, which may enhance the sensitivity and specificity. Noninvasive techniques for dynamically monitoring VAT can be further explored. Furthermore, future research should combine AP with other chronic inflammatory diseases to promote the clinical translation and intervention value of VAT-related indicators in AP.

This study represents the first meta-analysis specifically examining the association between VAT and AP. The findings indicate the independent prognostic significance of VAT, offering new insights into risk stratification and more individualized treatments for AP. Several limitations should be acknowledged. First and most importantly, the evidence base is constrained by significant methodological shortcomings in the included primary studies. All included studies were retrospective observational designs, which may introduce bias. Although subgroup analyses and meta-regression were performed to explore potential sources of heterogeneity, high heterogeneity among the studies was observed, and no sources of heterogeneity were identified. Critically, none of the included studies explicitly reported blinding of outcome assessors—a consistent and significant design weakness that may introduce detection bias, as knowledge of exposure status could theoretically influence outcome measurement or interpretation. Given that this limitation was universal across all included studies, its potential impact on the pooled estimates cannot be ruled out.Second, there may be differences in the methods used to measure VAT across studies, and the validity of different VAT measurement methods was not comparatively assessed. Furthermore, we only performed a subgroup analysis on the AUC results for VAI across different severity levels of AP, and did not further analyze the OR. We did not conduct subgroup analysis by different etiologies of AP. Third, due to the limited number of eligible studies, assessment of small-study effects and publication bias was not feasible, and a random-effects model was applied. Consequently, the results should be interpreted with caution and validated in future studies with larger sample sizes and standardized methodologies. Furthermore, in the subgroup analysis by regions, the studies from countries outside China were limited, with only one study available for each of these regions. As a result, the representativeness of these results is poor, and we did not discuss these studies in detail. Lastly, this study only examines VAT as a factor for AP, without considering its interaction with inflammatory biomarkers, which may play a role in mediating the association between VAT and AP. Additionally, we cannot speculate on the potential combined effects of VAT and inflammatory biomarkers, as these were not explored in the original studies. In light of these inherent limitations—particularly the universal lack of blinding and the observational design—the findings of this synthesis should be interpreted with caution. Future studies should prioritize blinding of outcome assessors and further explore the combined role of VAT and inflammatory biomarkers to enhance early risk assessment accuracy for AP.

## Conclusion

In conclusion, while this systematic review suggests that VAT is associated with SAP and demonstrates moderate discriminative ability for AP severity, these findings must be interpreted in light of significant methodological limitations. The available evidence, derived exclusively from observational studies with universal lack of blinding and substantial heterogeneity, does not allow any causal inferences. VAT measurement showed discriminative performance comparable to established tools such as SIRS and APACHE II in the included studies, and patients with more severe AP tended to have higher VAT levels. These observations support the hypothesis that VAT may contribute to AP progression and could be useful for guiding future risk stratification strategies. However, given the low certainty of the current evidence, these preliminary findings need confirmation in well-designed prospective cohort studies with rigorous blinding before any clinical or translational recommendations can be justified.

##  Supplemental Information

10.7717/peerj.21254/supp-1 Supplemental Information 1 Sensitivity of MAP VS MSAP

10.7717/peerj.21254/supp-2 Supplemental Information 2 MAP VS MSAP by measurement method

10.7717/peerj.21254/supp-3 Supplemental Information 3 MAP VS MSAP by regional

10.7717/peerj.21254/supp-4 Supplemental Information 4 Forest of MAP to MSAP

10.7717/peerj.21254/supp-5 Supplemental Information 5 Sensitivity of MAP VS SAP

10.7717/peerj.21254/supp-6 Supplemental Information 6 MAP VS SAP by measurement method

10.7717/peerj.21254/supp-7 Supplemental Information 7 MAP VS SAP by regional

10.7717/peerj.21254/supp-8 Supplemental Information 8 Forest of MAP to SAP

10.7717/peerj.21254/supp-9 Supplemental Information 9 Sensitivity of MSAP VS SAP

10.7717/peerj.21254/supp-10 Supplemental Information 10 MSAP VS SAP by measurement method

10.7717/peerj.21254/supp-11 Supplemental Information 11 MSAP VS SAP by regional

10.7717/peerj.21254/supp-12 Supplemental Information 12 Forest of MSAP to SAP

10.7717/peerj.21254/supp-13 Supplemental Information 13 Sensitivity of MAP vs MSAP-SAP

10.7717/peerj.21254/supp-14 Supplemental Information 14 MAP VS MSAP-SAP by measurement method

10.7717/peerj.21254/supp-15 Supplemental Information 15 MAP VS MSAP-SAP by regional

10.7717/peerj.21254/supp-16 Supplemental Information 16 Foest of MAP to MSAP-SAP

10.7717/peerj.21254/supp-17 Supplemental Information 17 Sensitivity of MAP-SMAP VS SAP

10.7717/peerj.21254/supp-18 Supplemental Information 18 MAP-SMAP VS SAP by measure method

10.7717/peerj.21254/supp-19 Supplemental Information 19 MAP-SAMP VS SAP by regional

10.7717/peerj.21254/supp-20 Supplemental Information 20 Forest of MAP-MSAP to SAP

10.7717/peerj.21254/supp-21 Supplemental Information 21 Sensitivity of SAP(OR)

10.7717/peerj.21254/supp-22 Supplemental Information 22 SAP(OR) by measurement method

10.7717/peerj.21254/supp-23 Supplemental Information 23 SAP(OR) by regional

10.7717/peerj.21254/supp-24 Supplemental Information 24 Sensitivity of AUC

10.7717/peerj.21254/supp-25 Supplemental Information 25 AUC ct

10.7717/peerj.21254/supp-26 Supplemental Information 26 Prediction by diesase

10.7717/peerj.21254/supp-27 Supplemental Information 27 Search History

10.7717/peerj.21254/supp-28 Supplemental Information 28 Assessment of study quality

10.7717/peerj.21254/supp-29 Supplemental Information 29 The result of meta regression

10.7717/peerj.21254/supp-30 Supplemental Information 30 Descriptions of Outcomes Involving Odds Ratios (OR) Included in the Article

10.7717/peerj.21254/supp-31 Supplemental Information 31 Raw data

10.7717/peerj.21254/supp-32 Supplemental Information 32 PRISMA 2020 Checklist

## Additional Information and Declarations

### Data Availability

The following information was supplied regarding data availability: This is a Systematic Review/Meta-analysis.
