# Supplementary figures and images for: Visceral adipose tissue and acute pancreatitis: a systematic review and meta-analysis
Source: PeerJ. 2026 Jun 2;14:e21254. doi: 10.7717/peerj.21254 (PMC13239464; doi:10.7717/peerj.21254)

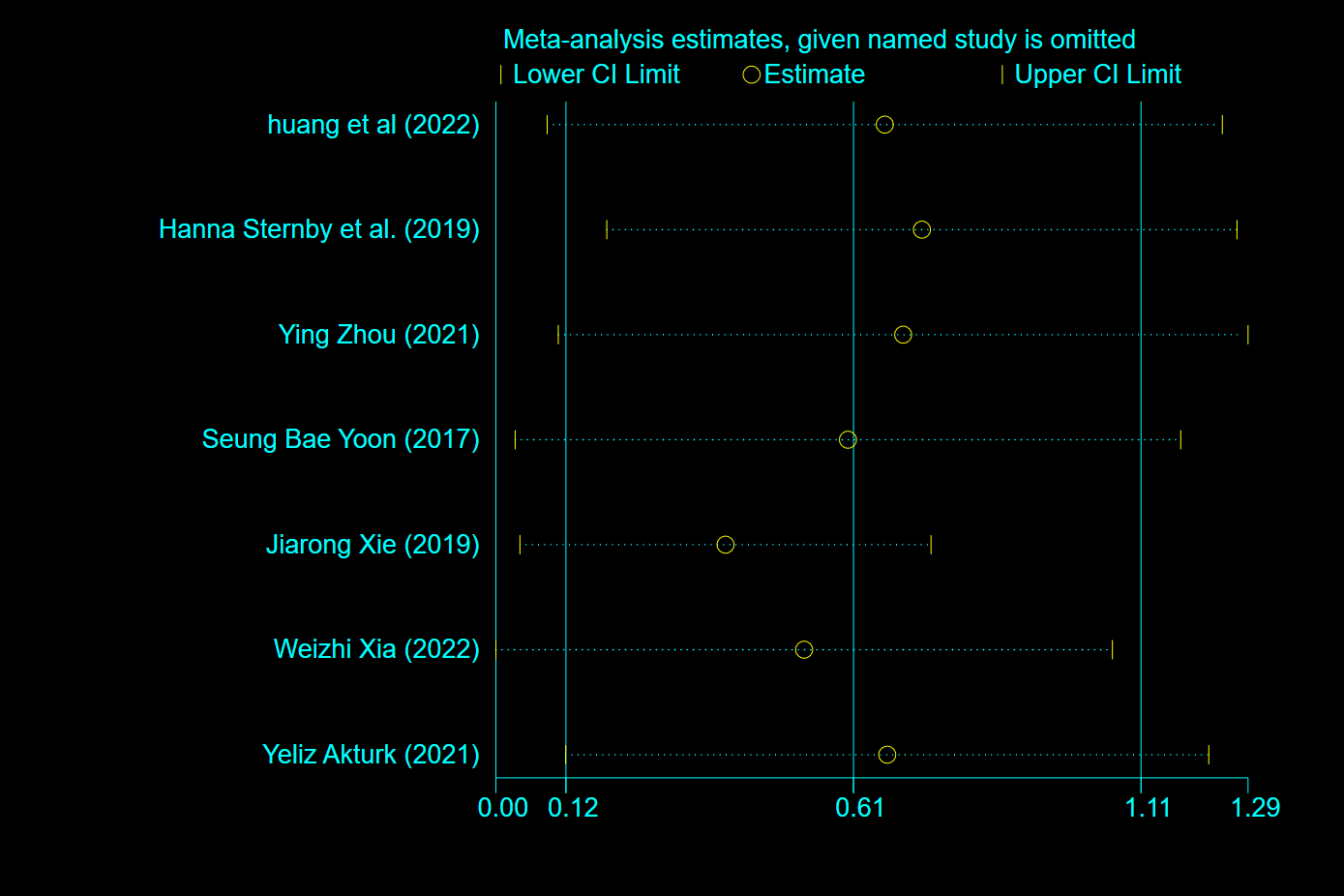

Supplement: Supplemental Information 1 [file peerj-14-21254-s001.png]

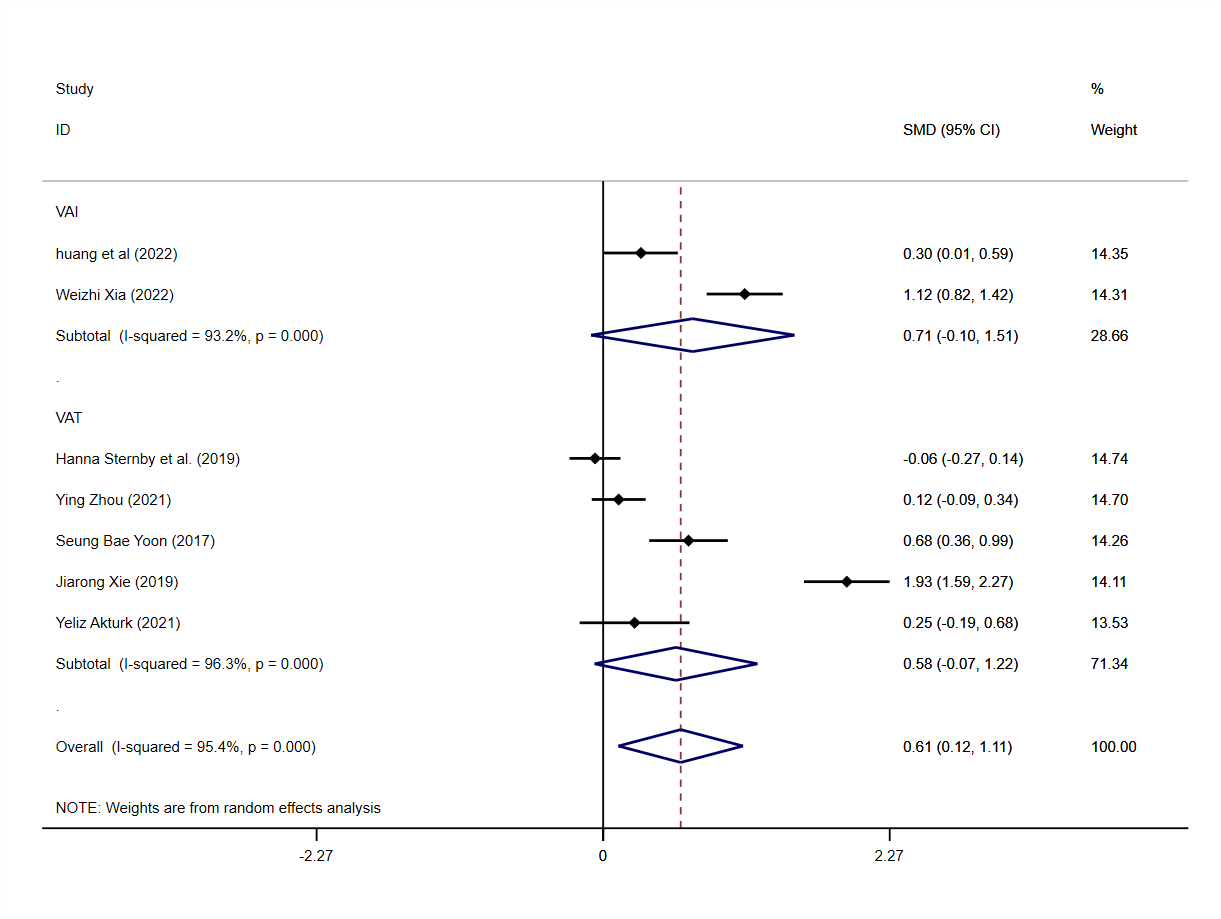

Supplement: Supplemental Information 2 [file peerj-14-21254-s002.png]

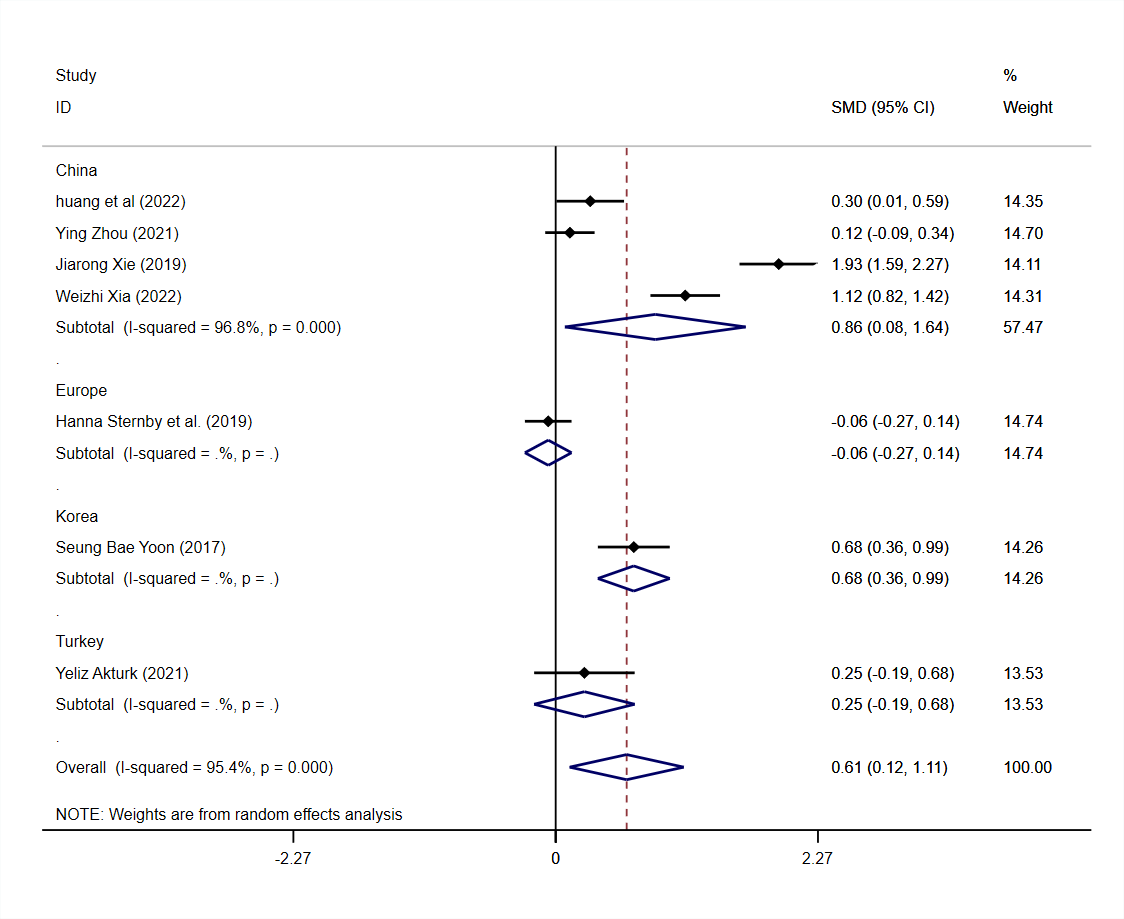

Supplement: Supplemental Information 3 [file peerj-14-21254-s003.png]

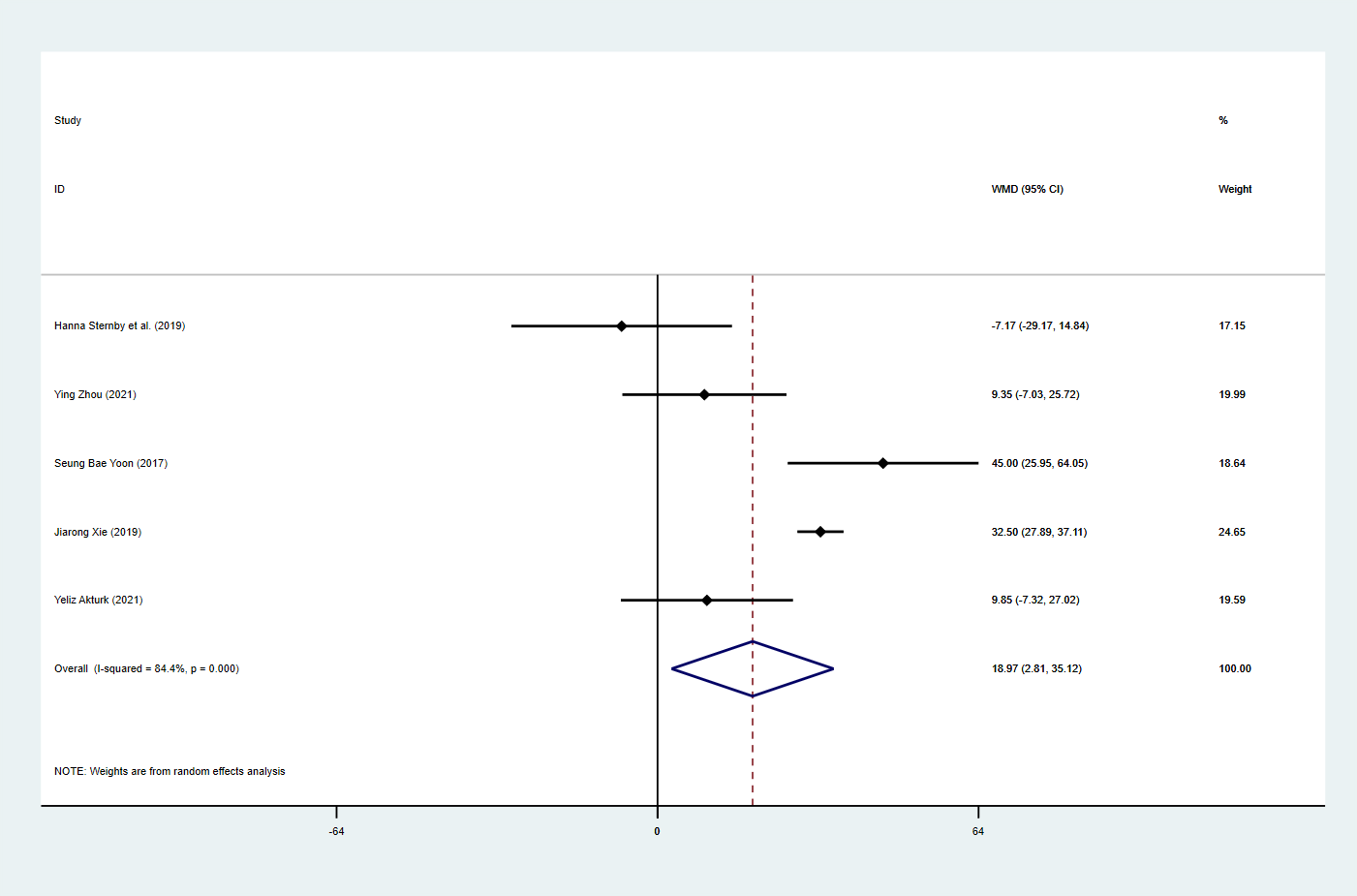

Supplement: Supplemental Information 4 [file peerj-14-21254-s004.png]

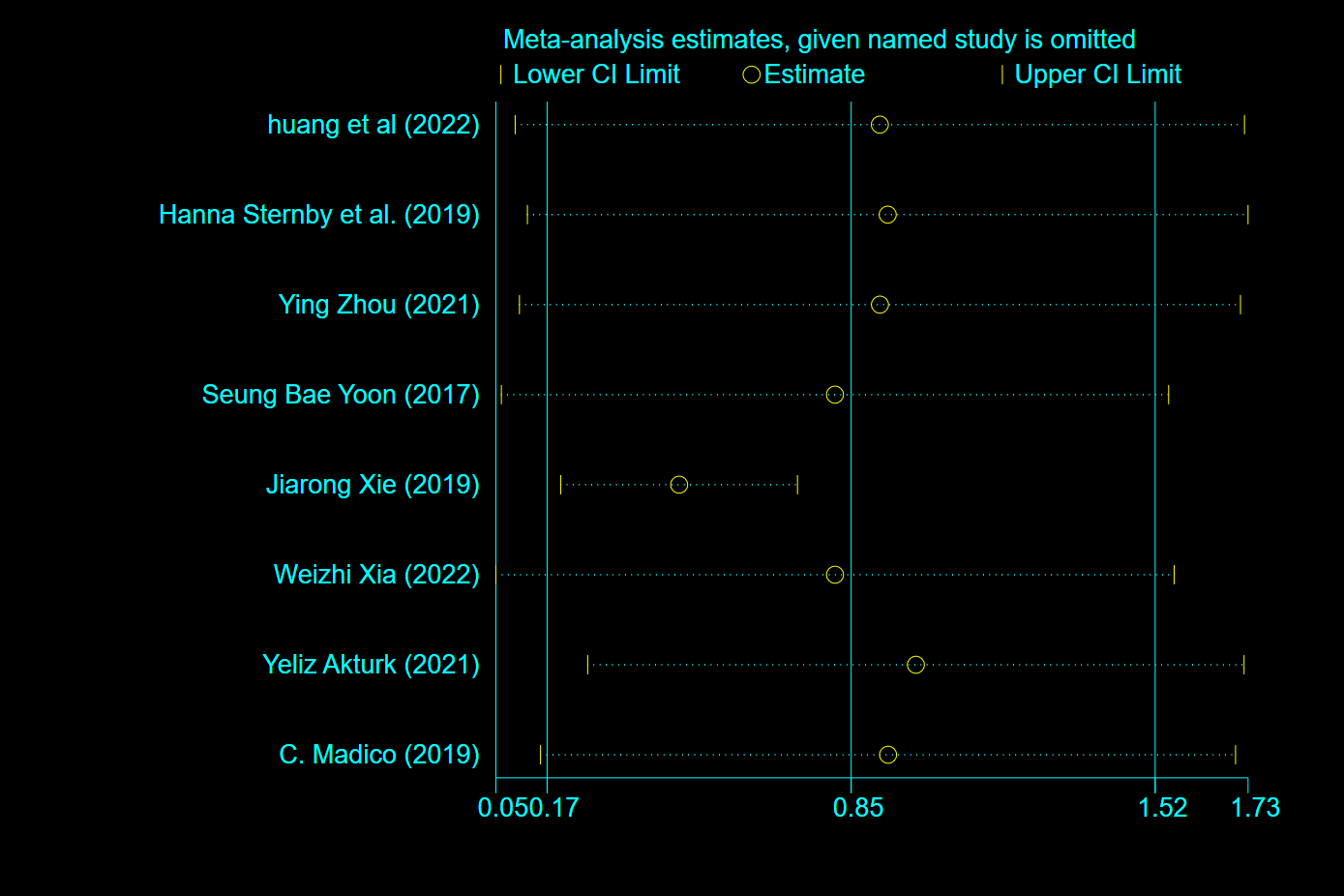

Supplement: Supplemental Information 5 [file peerj-14-21254-s005.png]

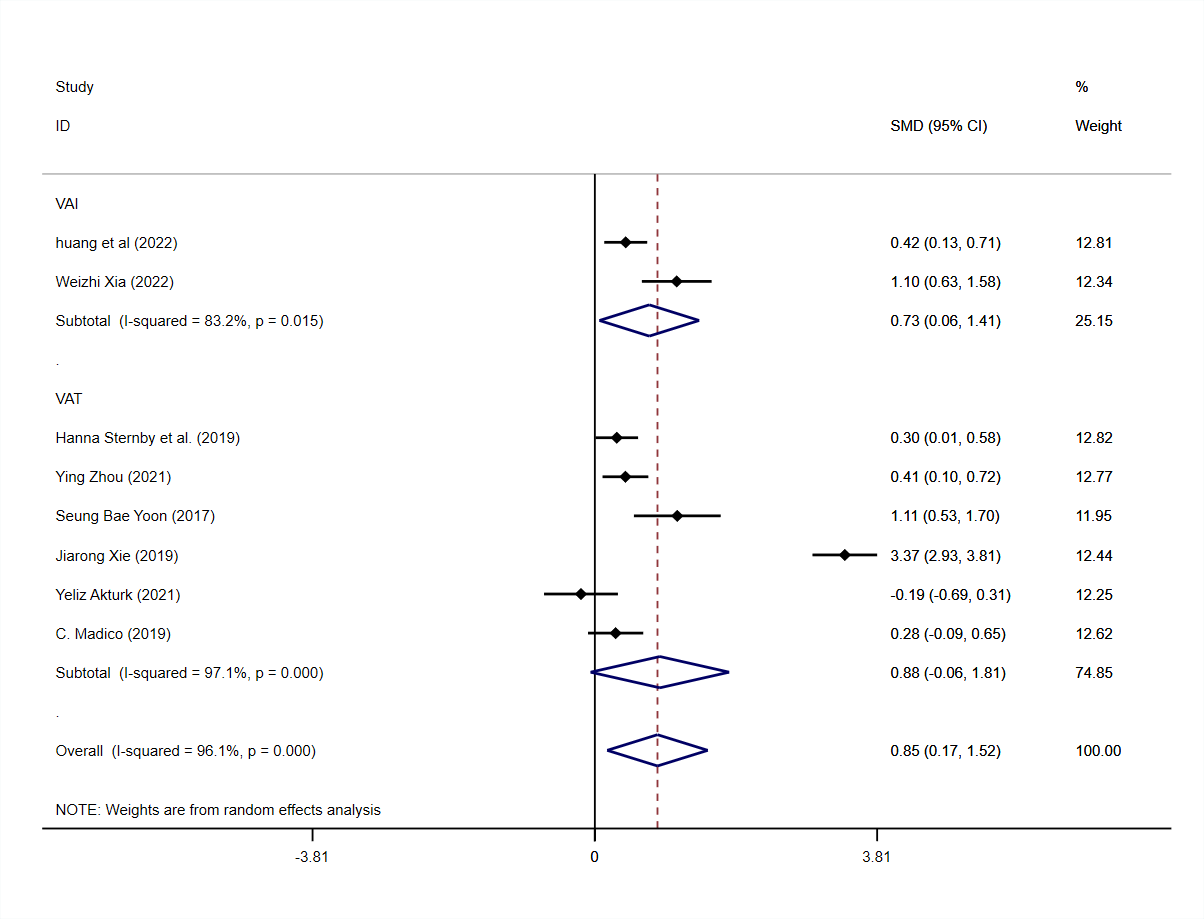

Supplement: Supplemental Information 6 [file peerj-14-21254-s006.png]

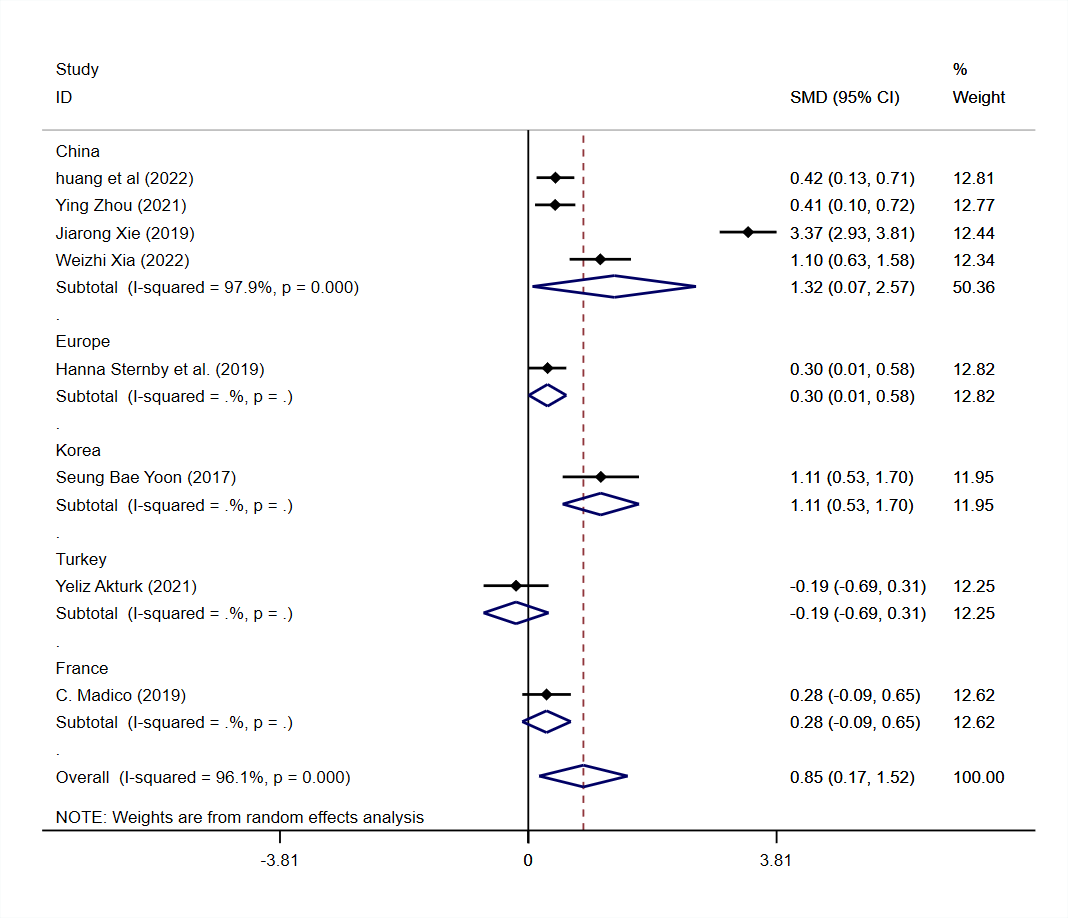

Supplement: Supplemental Information 7 [file peerj-14-21254-s007.png]

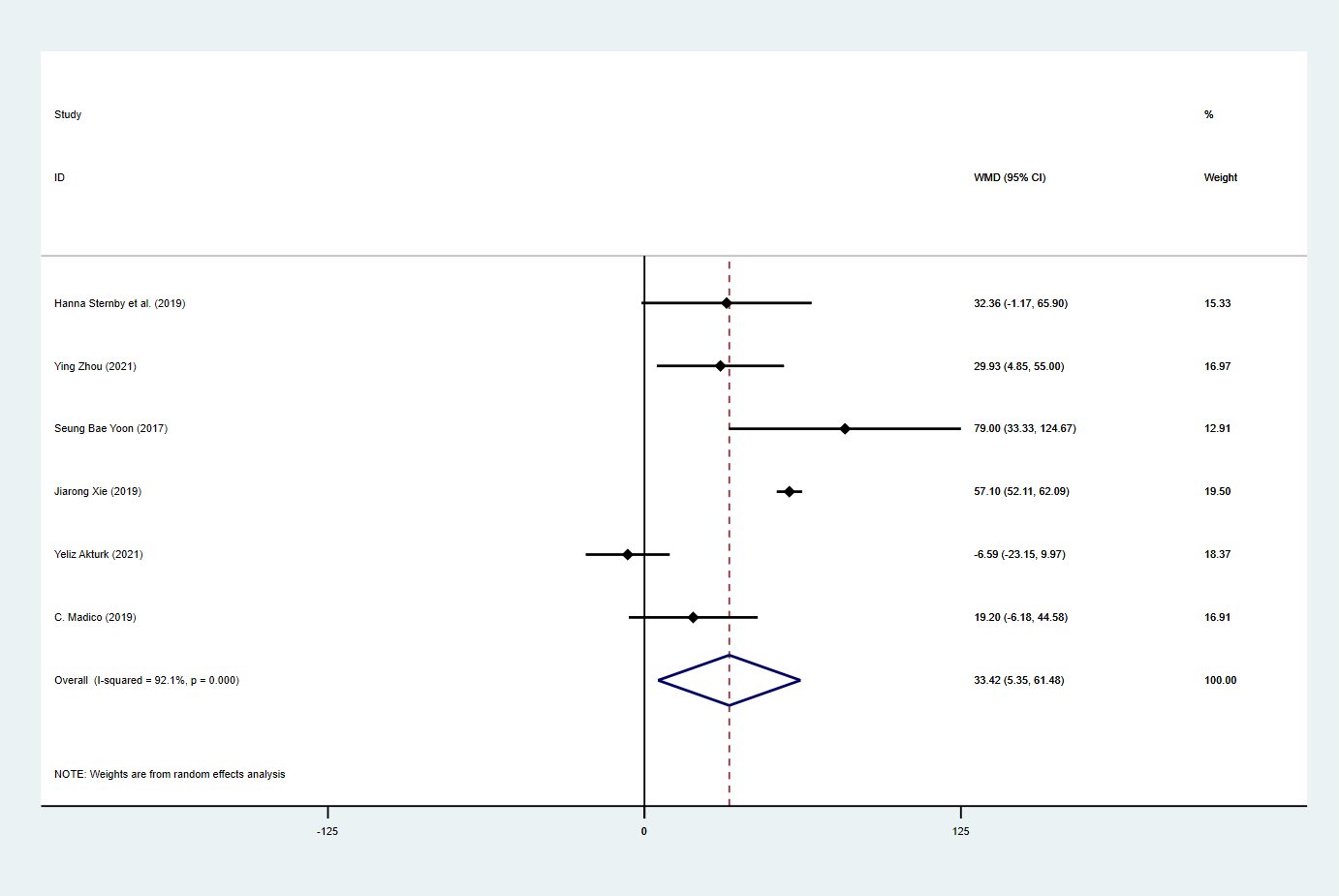

Supplement: Supplemental Information 8 [file peerj-14-21254-s008.png]

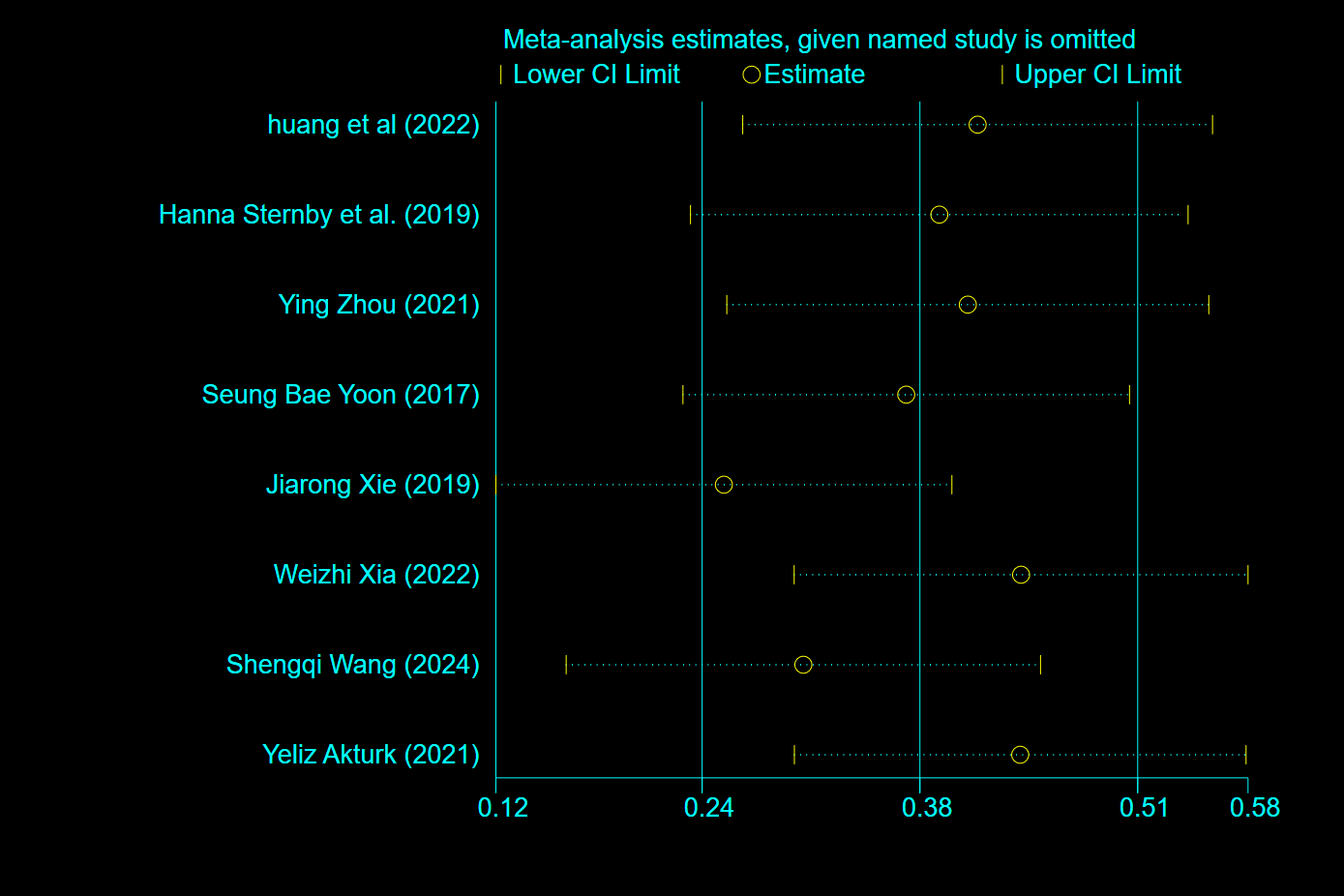

Supplement: Supplemental Information 9 [file peerj-14-21254-s009.png]

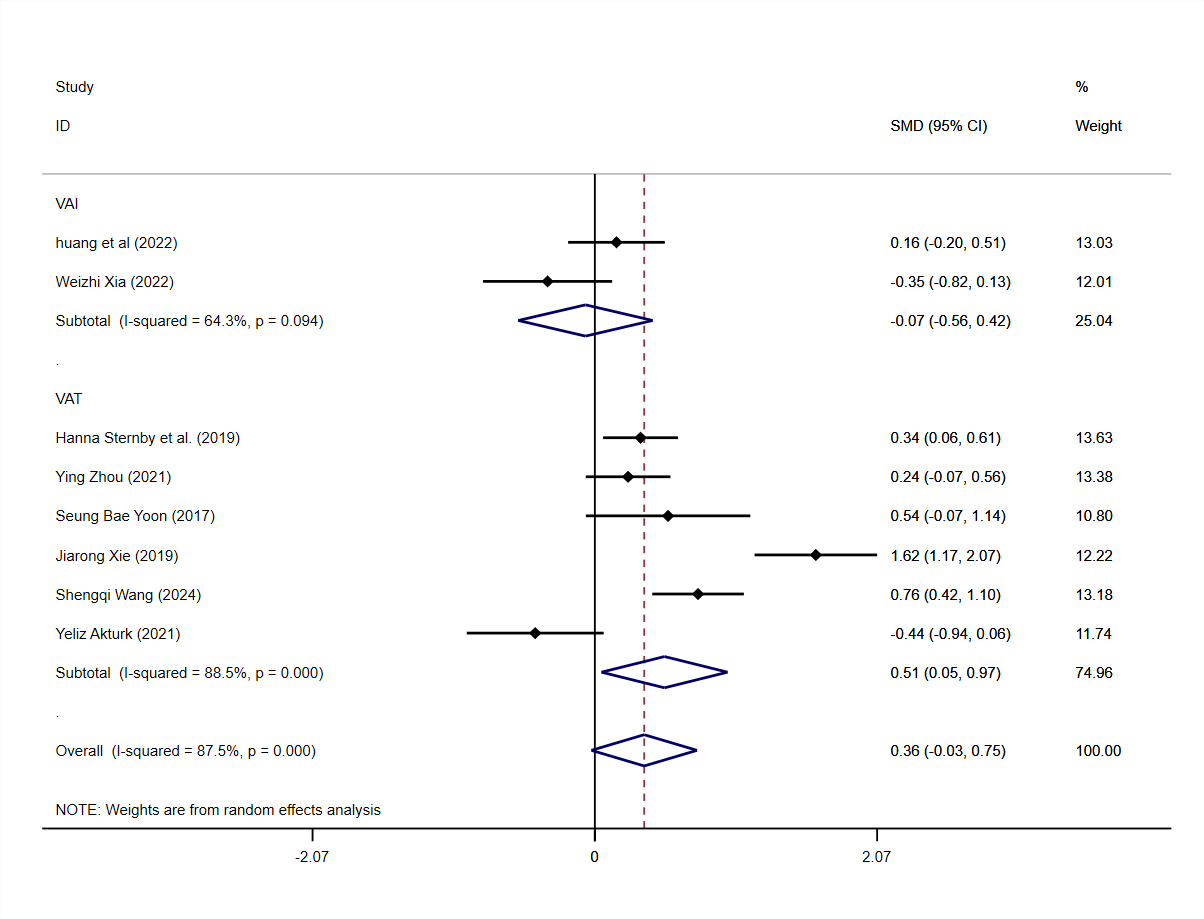

Supplement: Supplemental Information 10 [file peerj-14-21254-s010.png]

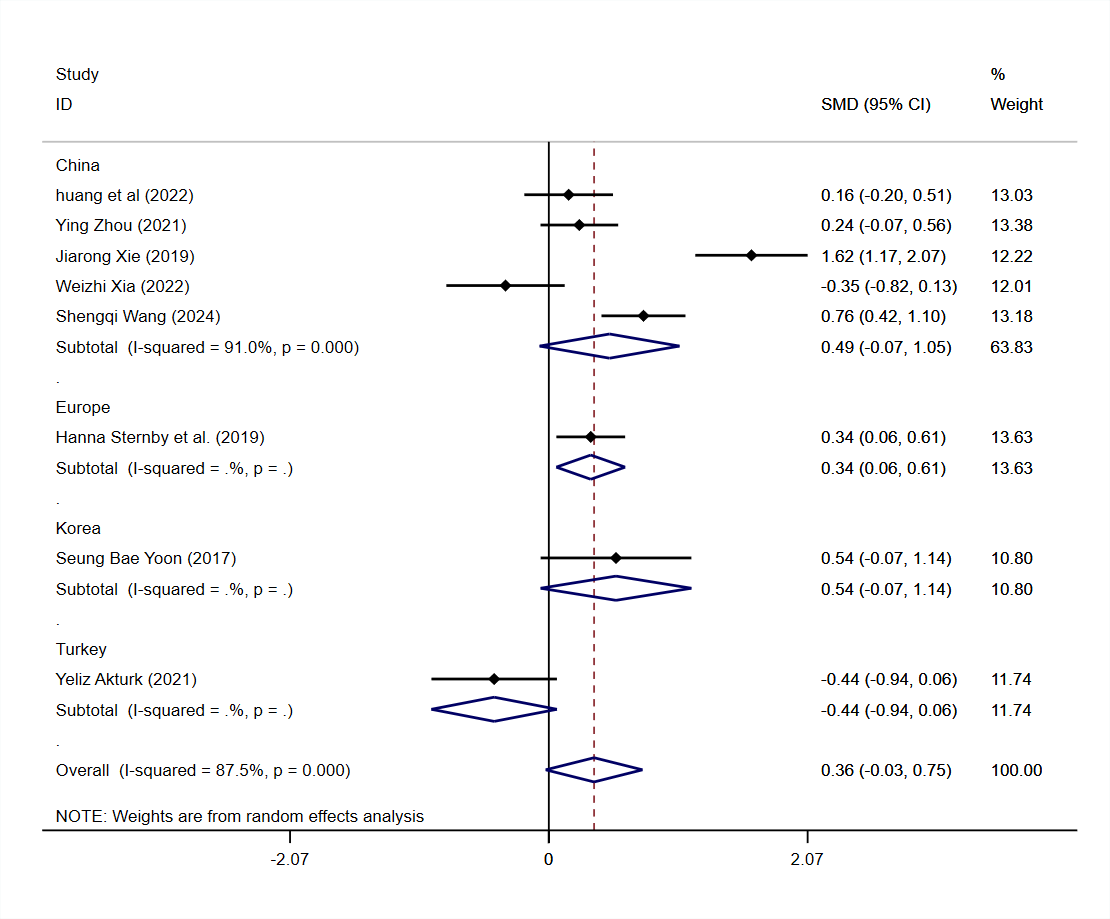

Supplement: Supplemental Information 11 [file peerj-14-21254-s011.png]

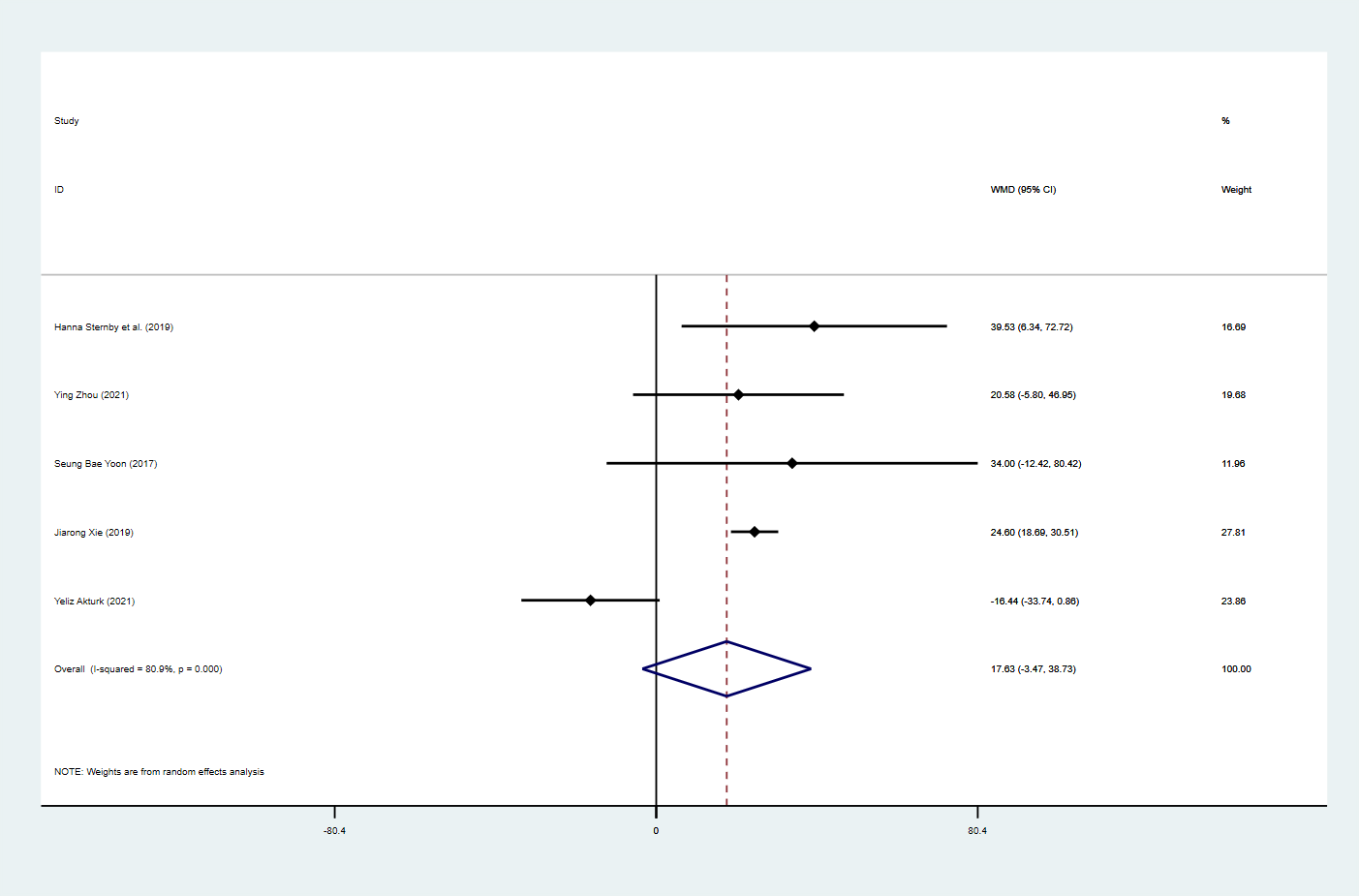

Supplement: Supplemental Information 12 [file peerj-14-21254-s012.png]

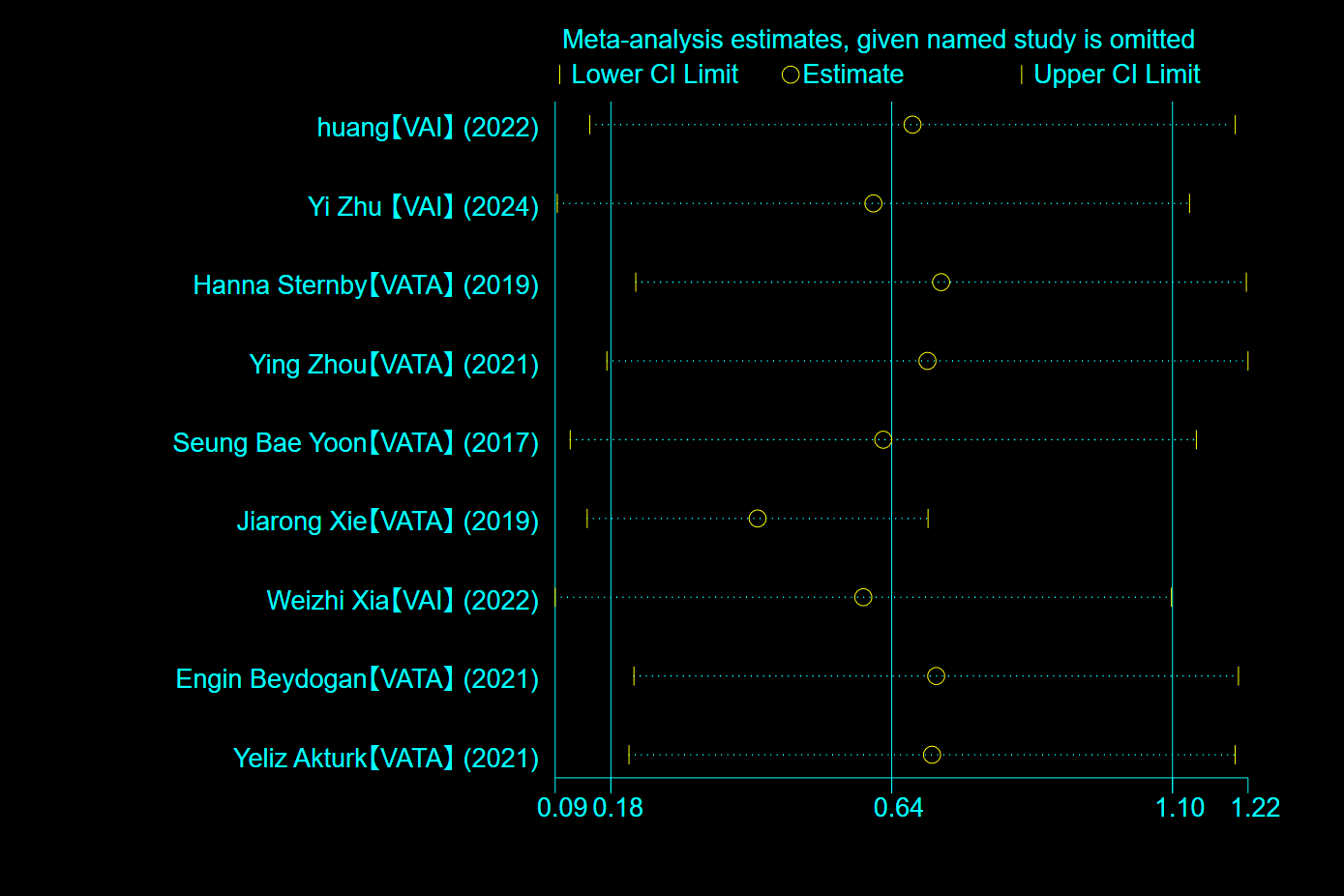

Supplement: Supplemental Information 13 [file peerj-14-21254-s013.png]

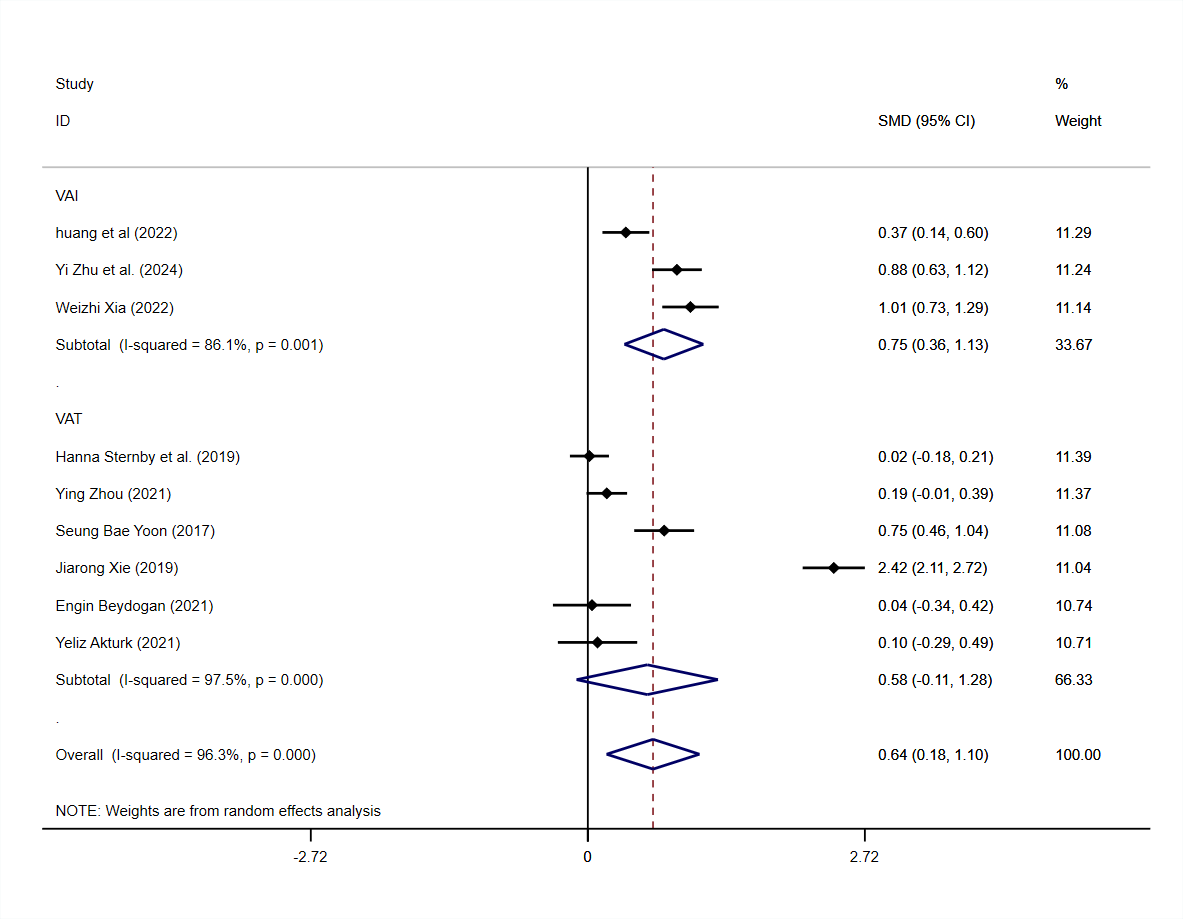

Supplement: Supplemental Information 14 [file peerj-14-21254-s014.png]

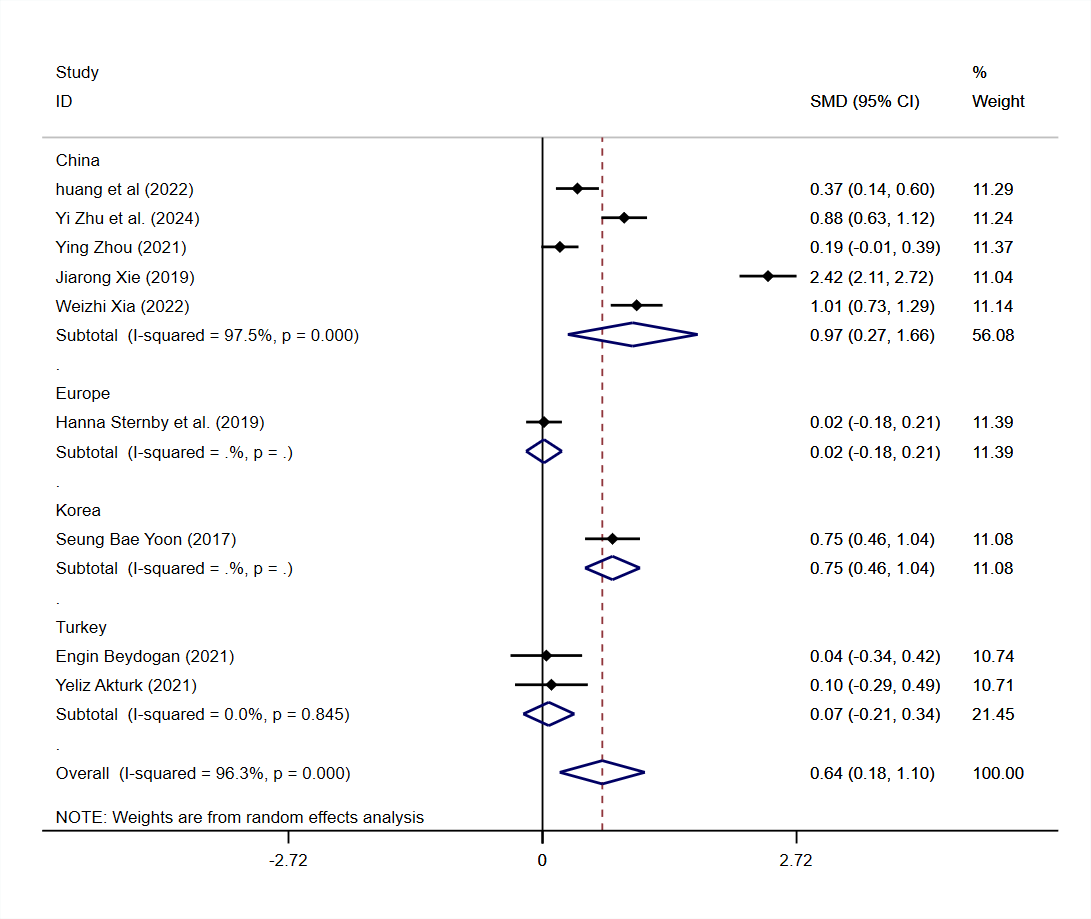

Supplement: Supplemental Information 15 [file peerj-14-21254-s015.png]

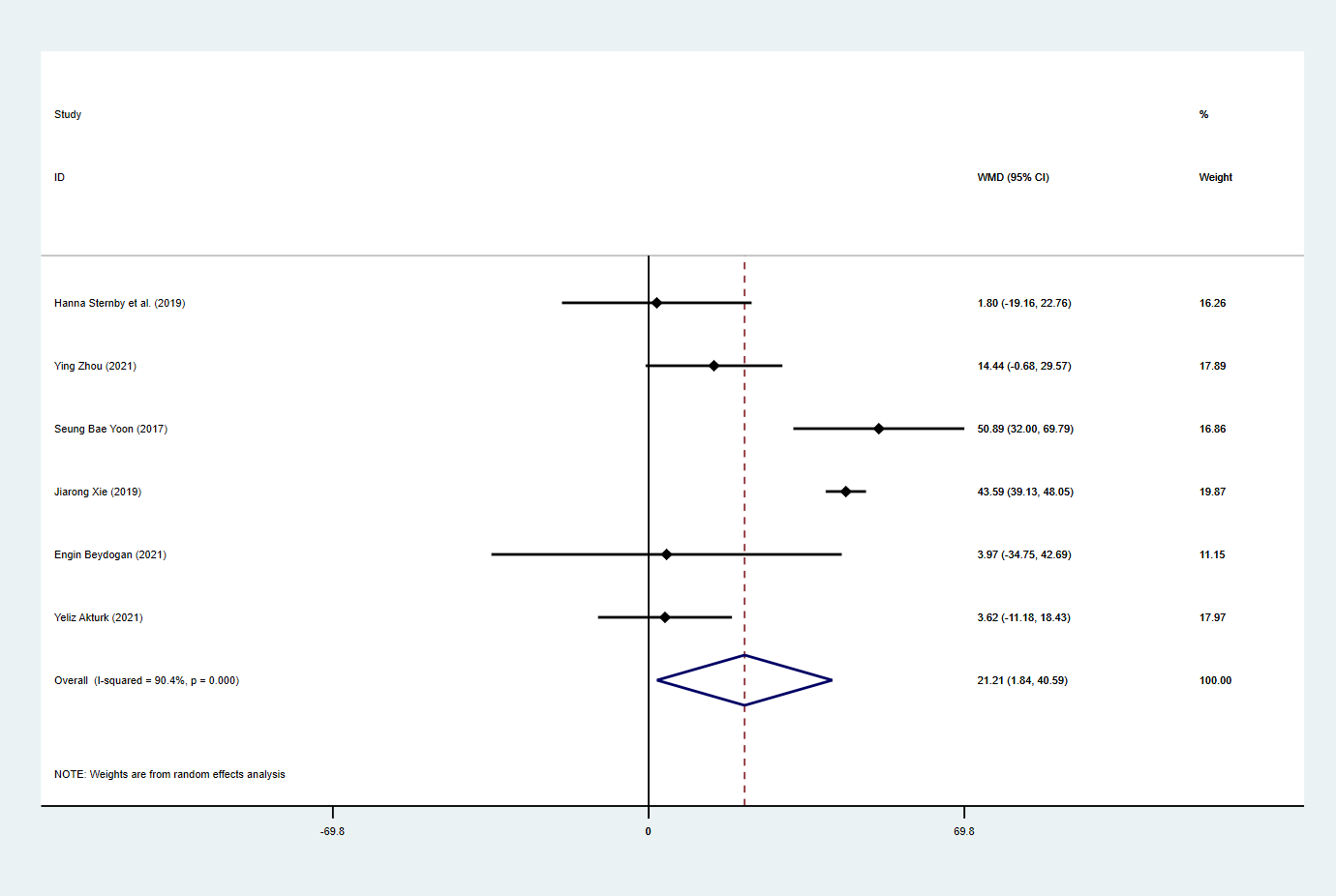

Supplement: Supplemental Information 16 [file peerj-14-21254-s016.png]

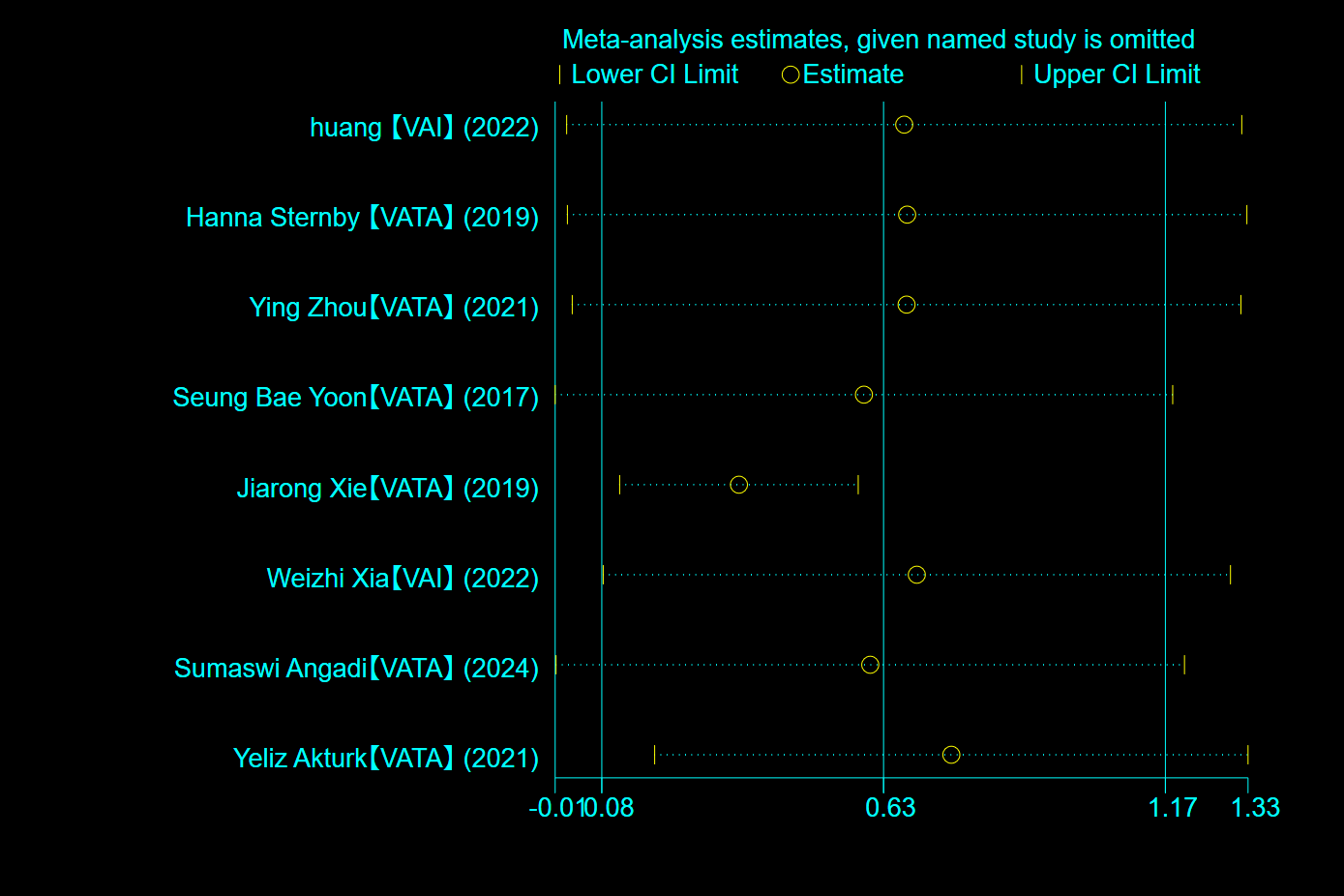

Supplement: Supplemental Information 17 [file peerj-14-21254-s017.png]

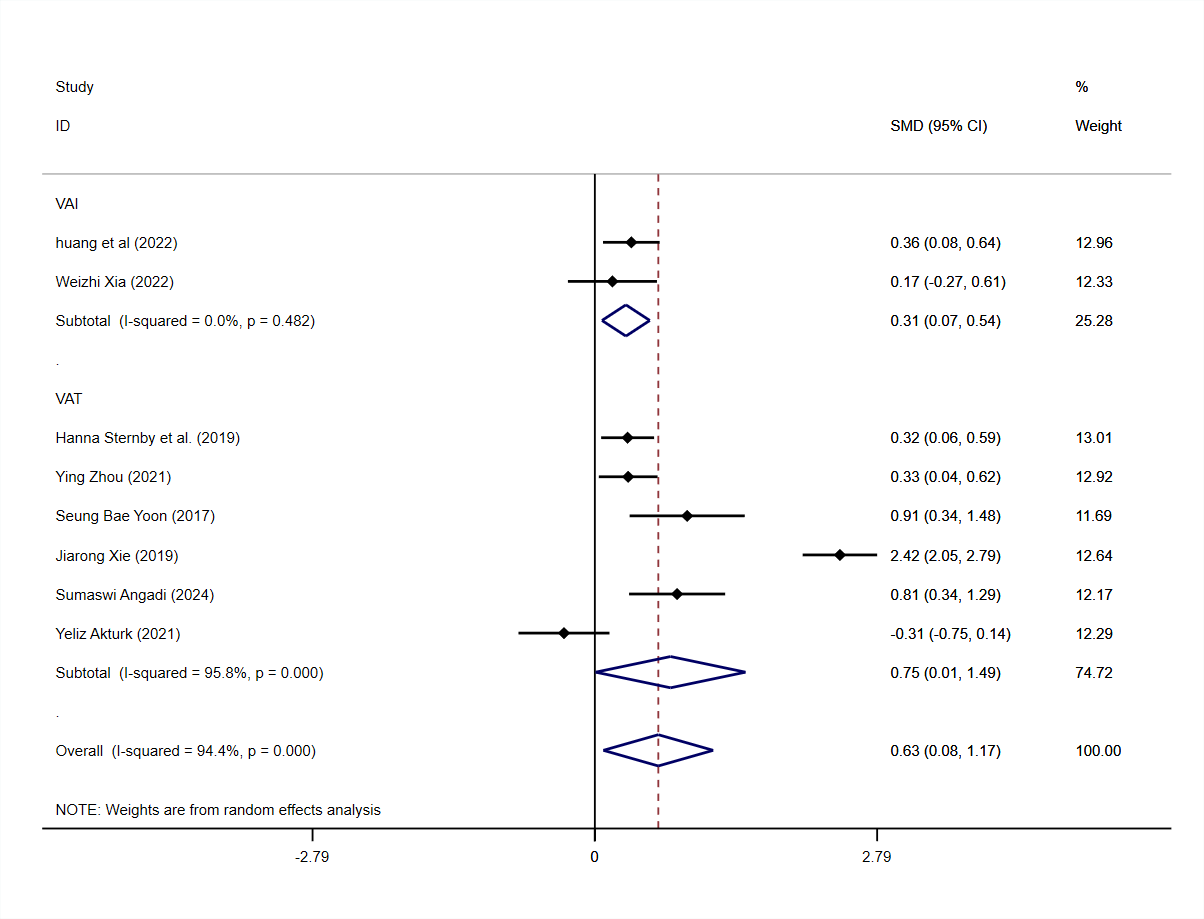

Supplement: Supplemental Information 18 [file peerj-14-21254-s018.png]

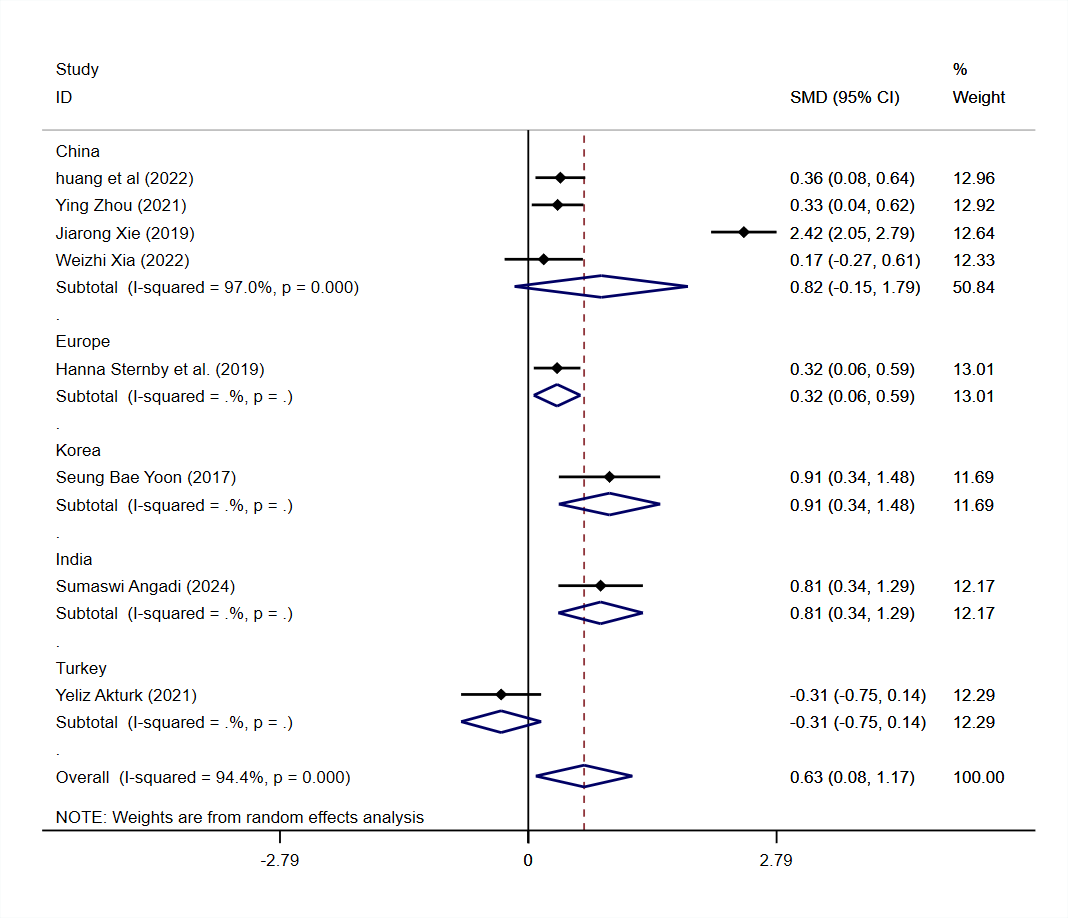

Supplement: Supplemental Information 19 [file peerj-14-21254-s019.png]

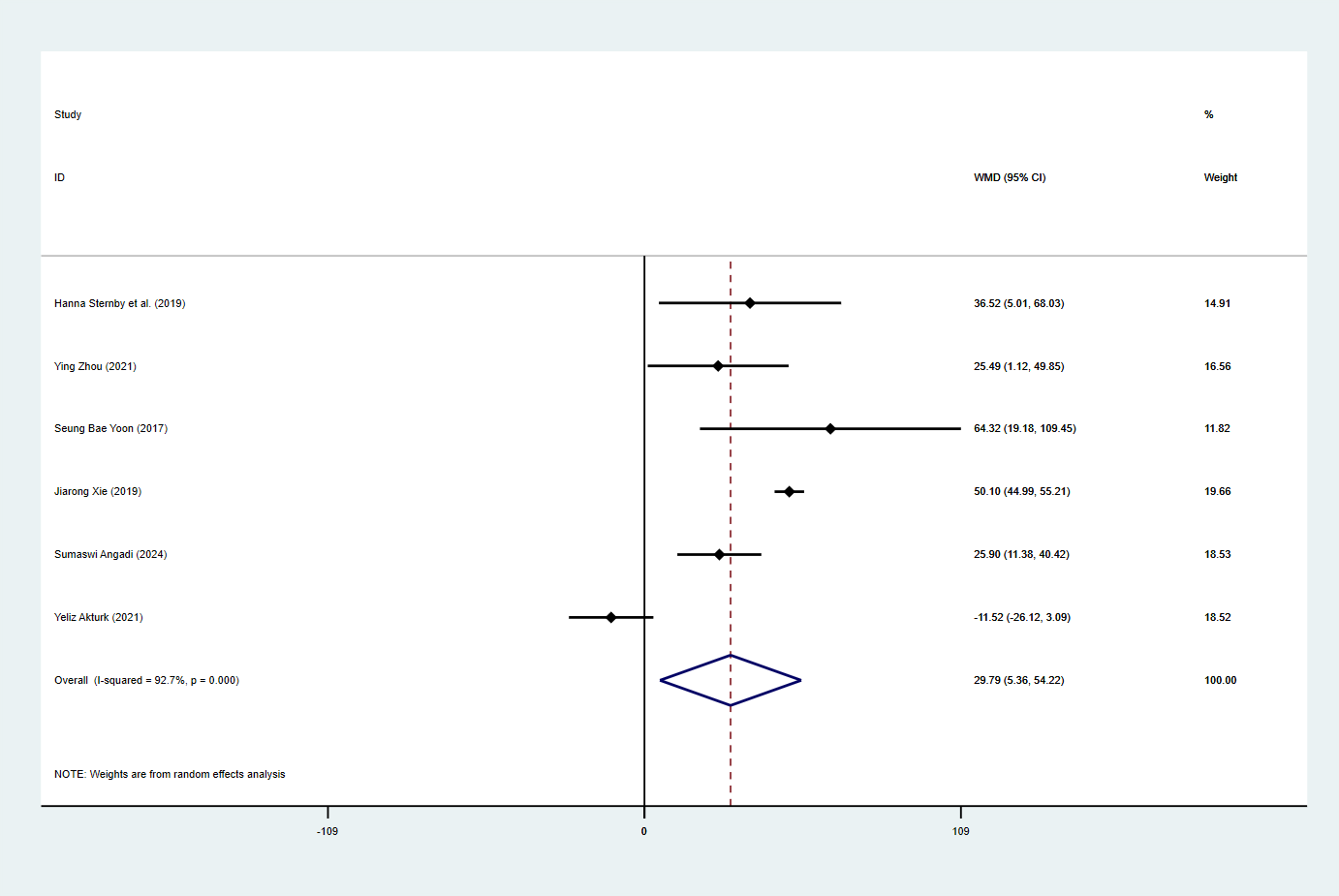

Supplement: Supplemental Information 20 [file peerj-14-21254-s020.png]

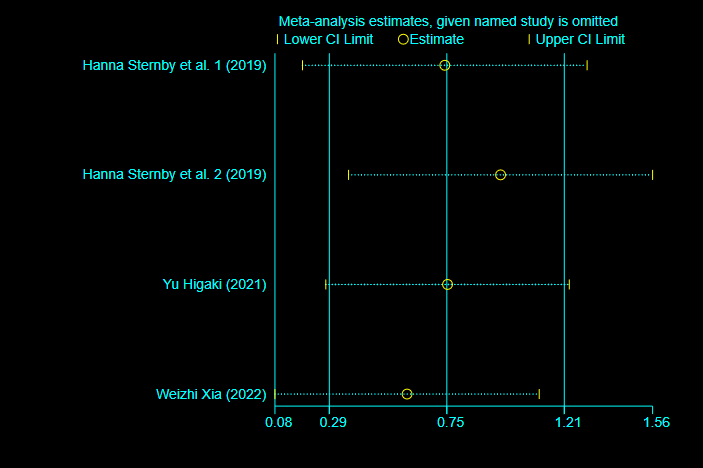

Supplement: Supplemental Information 21 [file peerj-14-21254-s021.png]

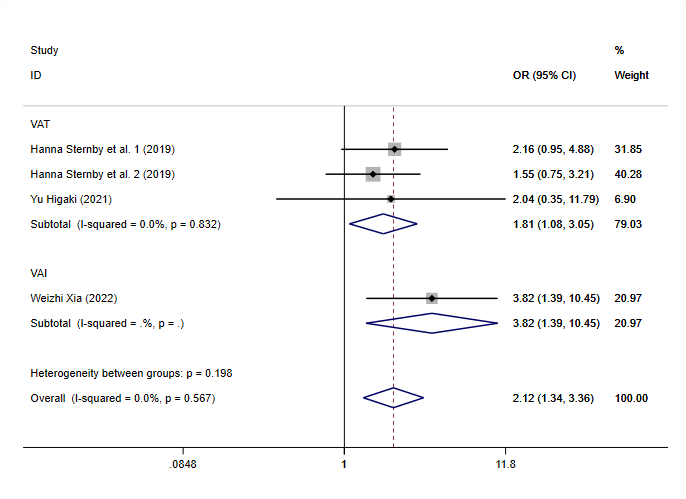

Supplement: Supplemental Information 22 [file peerj-14-21254-s022.png]

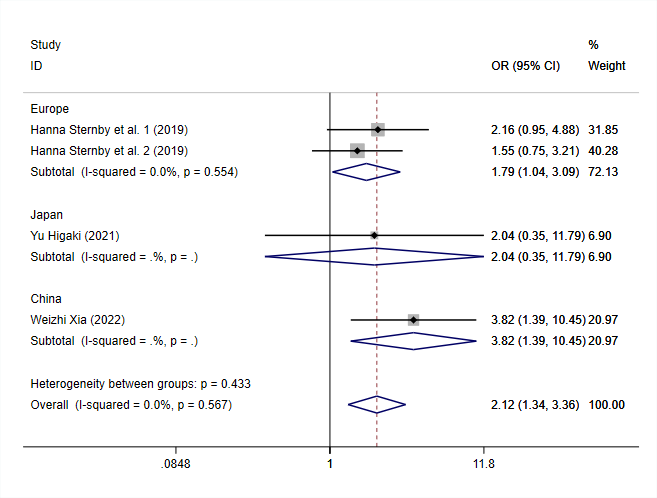

Supplement: Supplemental Information 23 [file peerj-14-21254-s023.png]

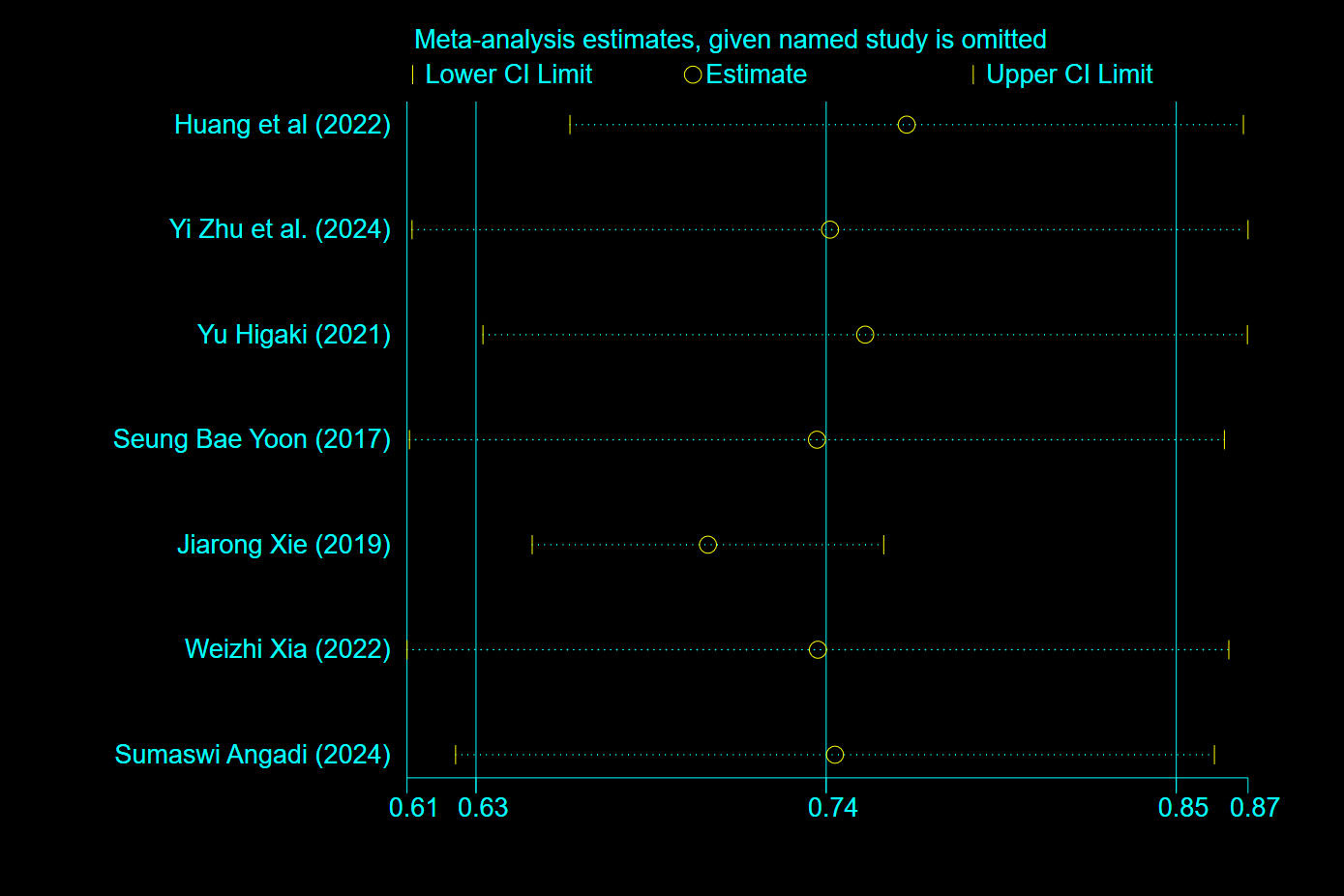

Supplement: Supplemental Information 24 [file peerj-14-21254-s024.png]

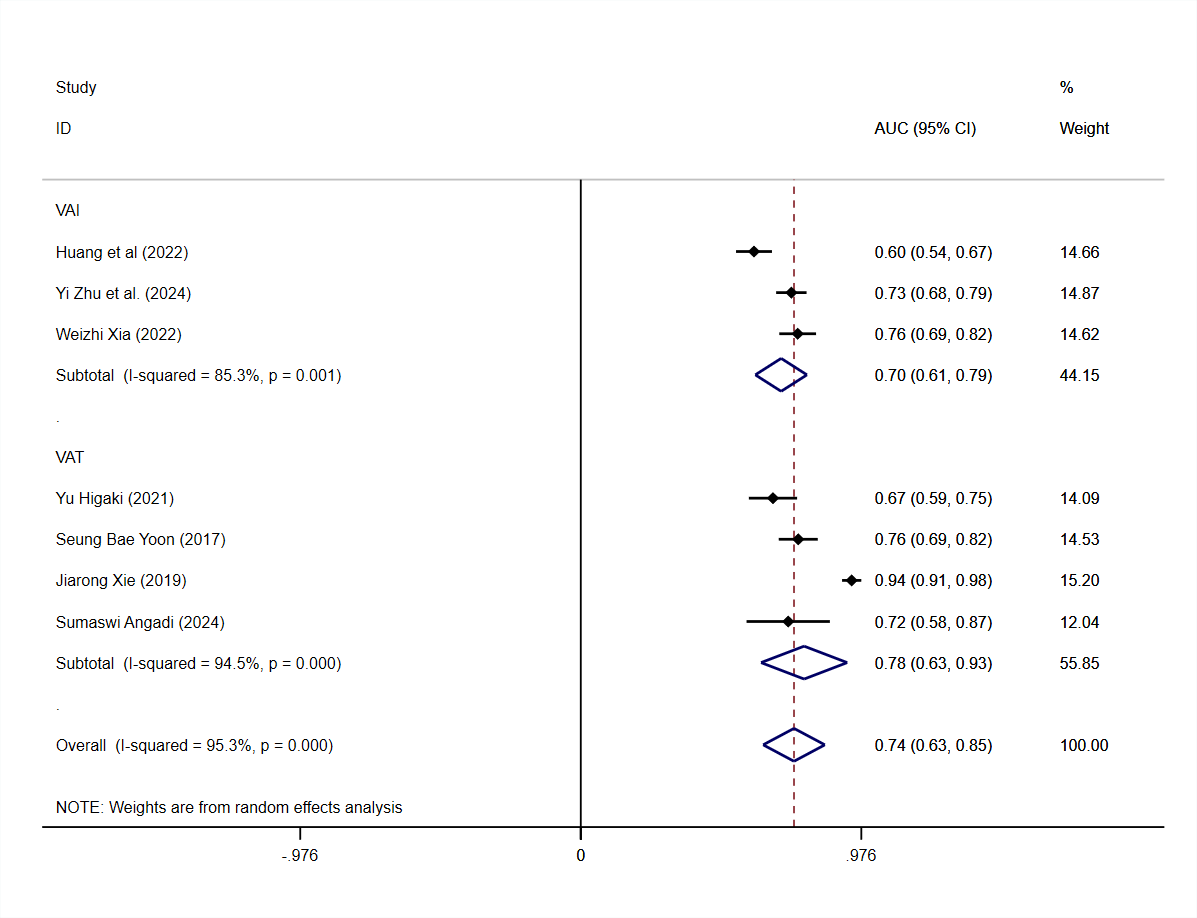

Supplement: Supplemental Information 25 [file peerj-14-21254-s025.png]

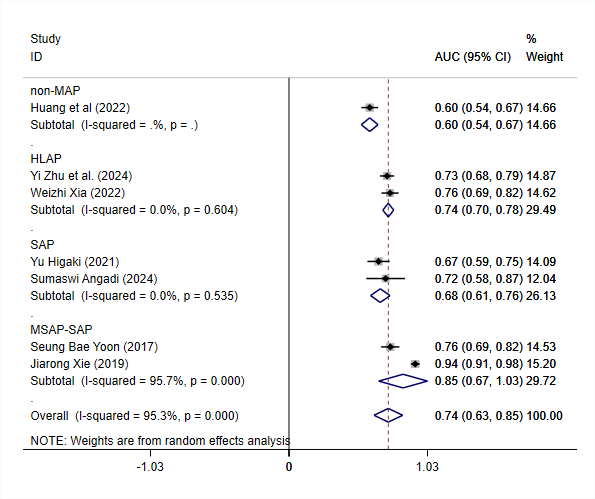

Supplement: Supplemental Information 26 [file peerj-14-21254-s026.png]
