# Supplementary material for: Visceral adipose tissue and acute pancreatitis: a systematic review and meta-analysis
Source: PeerJ. 2026 Jun 2;14:e21254. doi: 10.7717/peerj.21254 (PMC13239464; doi:10.7717/peerj.21254)
Supplement: Supplemental Information 27 [file peerj-14-21254-s027.docx]

Table S1 Search History

|  | Query | Results |
| --- | --- | --- |
| 1 | Pancreatitis [MeSH Terms] | 58,829 |
| 2 | ‘Acute Edematous Pancreatitides’[Title/Abstract] OR ‘Acute Edematous Pancreatitis’[Title/Abstract] OR ‘Acute Pancreatitides’[Title/Abstract] OR ‘Acute Pancreatitis’[Title/Abstract] OR ‘Edema, Pancreatic Parenchymal’[Title/Abstract] OR ‘Edematous Pancreatitides, Acute’[Title/Abstract] OR ‘Edematous Pancreatitis, Acute’[Title/Abstract] OR ‘Fat Necrosis, Peripancreatic’[Title/Abstract] OR ‘hereditary pancreatitis’[Title/Abstract] OR ‘Necrosis, Peripancreatic Fat’[Title/Abstract] OR ‘pancreas inflammation’[Title/Abstract] OR ‘pancreatic inflammation’[Title/Abstract] OR ‘Pancreatic Parenchyma with Edema’[Title/Abstract] OR ‘Pancreatic Parenchymal Edema’[Title/Abstract] OR ‘Pancreatic Parenchymal Edemas’[Title/Abstract] OR ‘Pancreatitides, Acute’[Title/Abstract] OR ‘Pancreatitides, Acute Edematous’[Title/Abstract] OR ‘pancreatitis’[Title/Abstract] OR ‘Pancreatitis, Acute’[Title/Abstract] OR ‘Pancreatitis, Acute Edematous’[Title/Abstract] OR ‘Parenchymal Edema, Pancreatic’[Title/Abstract] OR ‘Peripancreatic Fat Necroses’[Title/Abstract] OR ‘Peripancreatic Fat Necrosis’[Title/Abstract] OR ‘traumatic pancreatitis’[Title/Abstract] | 83,483 |
| 3 | Intra-Abdominal Fat[MeSH Terms] | 7,538 |
| 4 | ‘abdominal visceral adipose tissue’[Title/Abstract] OR ‘abdominal visceral fat’[Title/Abstract] OR ‘Abdominal Visceral Fats’[Title/Abstract] OR ‘Adipose Tissue, Intra-Abdominal’[Title/Abstract] OR ‘Adipose Tissue, Retroperitoneal’[Title/Abstract] OR ‘Adipose Tissue, Visceral’[Title/Abstract] OR ‘Fat, Abdominal Visceral’[Title/Abstract] OR ‘Fat, Intra Abdominal’[Title/Abstract] OR ‘Fat, Intra-Abdominal’[Title/Abstract] OR ‘Fat, Retroperitoneal’[Title/Abstract] OR ‘Fat, Visceral’[Title/Abstract] OR ‘Fats, Abdominal Visceral’[Title/Abstract] OR ‘Fats, Intra-Abdominal’[Title/Abstract] OR ‘Fats, Retroperitoneal’[Title/Abstract] OR ‘Fats, Visceral’[Title/Abstract] OR ‘Intra Abdominal Adipose Tissue’[Title/Abstract] OR ‘Intra Abdominal Fat’[Title/Abstract] OR ‘intraabdominal adipose tissue’[Title/Abstract] OR ‘intra-abdominal adipose tissue’[Title/Abstract] OR ‘intraabdominal fat’[Title/Abstract] OR ‘intra-abdominal fat’[Title/Abstract] OR ‘Intra-Abdominal Fats’[Title/Abstract] OR ‘organ adipose tissue’[Title/Abstract] OR ‘organ fat’[Title/Abstract] OR ‘Retroperitoneal Adipose Tissue’[Title/Abstract] OR ‘Retroperitoneal Fat’[Title/Abstract] OR ‘Retroperitoneal Fats’[Title/Abstract] OR ‘visceral abdominal adipose tissue’[Title/Abstract] OR ‘visceral abdominal fat’[Title/Abstract] OR ‘visceral adipose tissue’[Title/Abstract] OR ‘visceral fat’[Title/Abstract] OR ‘Visceral Fats’[Title/Abstract] | 28,593 |
| 5 | (#1 OR #2) AND (#3 OR #4) | 219 |
