# Supplementary material for: Visceral adipose tissue and acute pancreatitis: a systematic review and meta-analysis
Source: PeerJ. 2026 Jun 2;14:e21254. doi: 10.7717/peerj.21254 (PMC13239464; doi:10.7717/peerj.21254)
Supplement: Supplemental Information 29 [file peerj-14-21254-s029.docx]

Table S3 The result of meta regression

|  | Variable | P value |
| --- | --- | --- |
| MAP vs MSAP | Measurement index | 0.844 |
|  | Ages | 0.483 |
|  | BMI | 0.304 |
| MAP vs SAP | Measurement index | 0.829 |
|  | Ages | 0.714 |
|  | BMI | 0.324 |
| MSAP vs SAP | Measurement index | 0.289 |
|  | Ages | 0.751 |
|  | BMI | 0.470 |
| MAP vs MSAP-SAP | Measurement index | 0.779 |
|  | Ages | 0.395 |
|  | BMI | 0.889 |
| MAP-MSAP vs SAP | Measurement index | 0.516 |
|  | Ages | 0.923 |
|  | BMI | 0.236 |

Note: BMI: body mass index; MAP: mild acute pancreatitis; MSAP: moderately severe acute pancreatitis; SAP: severe acute pancreatitis.
