# Supplementary material for: Visceral adipose tissue and acute pancreatitis: a systematic review and meta-analysis
Source: PeerJ. 2026 Jun 2;14:e21254. doi: 10.7717/peerj.21254 (PMC13239464; doi:10.7717/peerj.21254)
Supplement: Supplemental Information 30 [file peerj-14-21254-s030.docx]

Table S4 Descriptions of Outcomes Involving Odds Ratios (OR) Included in the Article

| Author | Year | Group | Outcome | Adjust Factor | Cut Off |
| --- | --- | --- | --- | --- | --- |
| Huang | 2022 | No-Lower Vs Lower | Non - Mild Acute Pancreatitis | Cmi、Lap、Wti、Cvai、Wc | 79.633 |
| Hanna Sternby | 2019 | Highest Tertile Vs Lowest Tertile | Severity Acute Pancreatitis. | Age; Gender | NI |
|  |  | Mid Tertile Vs Lowest Tertile |  |  |  |
| Yu Higaki | 2021 | No-Lower Vs Lower | Severe Acute Pancreatitis | Prognostic Factor Score;Age | 167 |
| Ying Zhou | 2021 | Each Additional Unit Of Area（Cm^2^） | Severe Acute Pancreatitis Or Severe Acute Pancreatitis | Smoking; Drinking; Diabetes; Previous Ap History; L3-Smi; Smt; Sata; Sat; Vata; Bmi; Sex;Age |  |
| Weizhi Xia | 2022 | No-Lower Vs Lower | Hyperlipidaemia Acute Pancreatitis | Alb; Tg; Tc | 35.11 |
